# Structural insights into human exon-defined spliceosome prior to activation

**DOI:** 10.1038/s41422-024-00949-w

**Published:** 2024-04-24

**Authors:** Wenyu Zhang, Xiaofeng Zhang, Xiechao Zhan, Rui Bai, Jianlin Lei, Chuangye Yan, Yigong Shi

**Affiliations:** 1 Beijing Frontier Research Center for Biological Structure, Tsinghua-Peking Joint Center for Life Sciences, School of Life Sciences, Tsinghua University, Beijing, China; 2 Key Laboratory of Structural Biology of Zhejiang Province, School of Life Sciences, Westlake University, Hangzhou, Zhejiang China; 3 Westlake Laboratory of Life Sciences and Biomedicine, Hangzhou, Zhejiang China; 4 Institute of Biology, Westlake Institute for Advanced Study, Hangzhou, Zhejiang China

**Keywords:** Cryoelectron microscopy, Ribozymes

## Abstract

Spliceosome is often assembled across an exon and undergoes rearrangement to span a neighboring intron. Most states of the intron-defined spliceosome have been structurally characterized. However, the structure of a fully assembled exon-defined spliceosome remains at large. During spliceosome assembly, the pre-catalytic state (B complex) is converted from its precursor (pre-B complex). Here we report atomic structures of the exon-defined human spliceosome in four sequential states: mature pre-B, late pre-B, early B, and mature B. In the previously unknown late pre-B state, U1 snRNP is already released but the remaining proteins are still in the pre-B state; unexpectedly, the RNAs are in the B state, with U6 snRNA forming a duplex with 5′-splice site and U5 snRNA recognizing the 3′-end of the exon. In the early and mature B complexes, the B-specific factors are stepwise recruited and specifically recognize the exon 3′-region. Our study reveals key insights into the assembly of the exon-defined spliceosomes and identifies mechanistic steps of the pre-B-to-B transition.

## Introduction

**Fig. 1:**
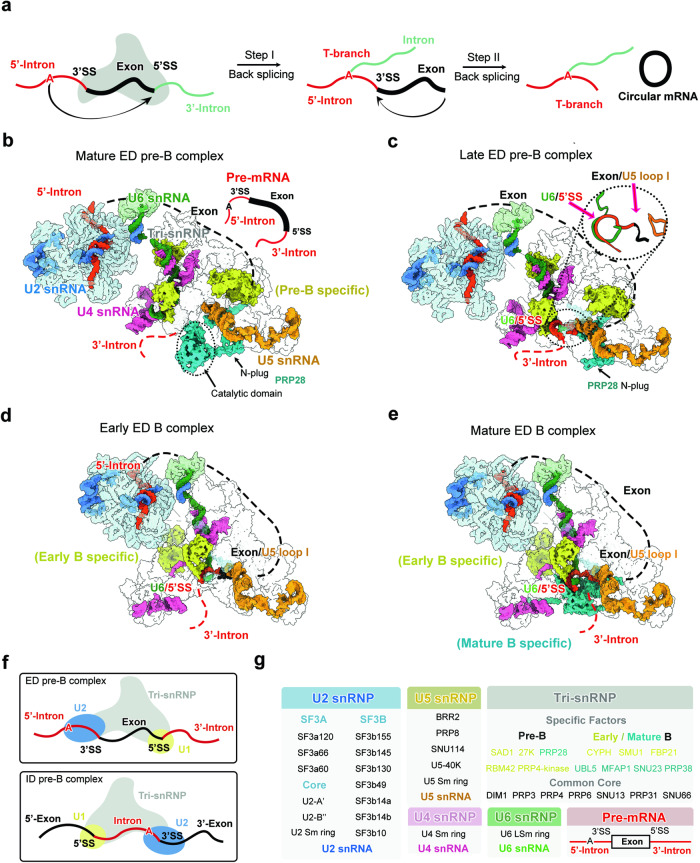
Cryo-EM structures of the human ED pre-B and B complexes. **a** A schematic diagram of back-splicing. The pre-mRNA shown here only contains an exon that is bracketed by two partial introns. The T-branched RNA intermediate is the hallmark of back-splicing. **b** Overall structure of the human mature ED pre-B complex. This and the other three spliceosome structures are displayed in surface representation, with snRNAs and select protein components color-coded. Intron and exon of the pre-mRNA are colored red and black, respectively. **c** Overall structure of the human late ED pre-B complex. The close-up view highlights the U6/5′SS duplex and U5 loop I/exon duplex. **d** Overall structure of the human early ED B complex. **e** Overall structure of the human mature ED B complex. **f** Cartoon diagrams of the ED pre-B complex (upper panel) and ID pre-B complex (lower panel). **g** Tabulation of the spliceosomal components that have been identified in the four cryo-EM reconstructions of human ED spliceosome.

In vertebrates, especially human, the average length of exons is considerably shorter than that of introns.^1–3^ The spliceosome tends to assemble over these relatively short exons.^2^ The exon-defined (ED) spliceosomes are often converted to intron-defined (ID) spliceosomes prior to their activation, as introns must be properly excised to generate mRNA.^2,4–6^ In cells, back-splicing by the ED spliceosomes may also proceed to produce a characteristic T-branched RNA intermediate, which further generates a circular exon as the final product (Fig. 1a).

The human ID spliceosome prior to activation has been structurally characterized in atomic details.^6–14^ U1 and U2 small nuclear ribonucleoproteins (snRNPs) recognize the 5′-splice site (5′SS) and the branch point sequence (BPS), respectively, to form the pre-spliceosome (known as the A complex).^15–17^ The A complex associates with U4/U6.U5 tri-snRNP to form the pre-B complex, in which U2 snRNP is connected to tri-snRNP mainly through the U2/U6 duplex.^9,11,18^ The RNA helicase PRP28 unwinds the U1/5′SS duplex,^19^ releasing U1 snRNP and allowing formation of the U6/5′SS duplex in the B complex. The pre-B-to-B remodeling involves a pronounced structural reorganization.^11^

In contrast to the ID state, structural information on the ED spliceosome is scarce and only available for early assemblies prior to the A complex.^6,20,21^ Our understanding of the ED state is largely based on biochemical analysis.^5,6,22–25^ In the A complex of the ED state, U1 and U2 snRNPs on two ends of the exon are thought to be bridged by the SR proteins in the middle.^24–26^ In the B complex of the ED state, tri-snRNP association could be stabilized by exogenous 5′SS oligonucleotides.^5,22^ In addition, endogenous circular mRNA is present in the purified yeast post-catalytic spliceosome (the P complex) of the ED state,^23^ confirming the back-splicing pathway^6,27^ (Fig. 1a).

The ID and ED complexes share an identical set of snRNPs.^5,6,25^ The ED-to-ID conversion may occur right after formation of the A state, as tri-snRNP may associate with U1 and U2 snRNPs that span an intron to form the ID pre-B complex.^2,4^ Alternatively, the ED-to-ID conversion may also take place at the B state, where the 5′SS of the upstream intron invades the B complex of the ED state to displace the 5′SS of the downstream intron.^5,6^ The mechanism of the ED-to-ID conversion has remained enigmatic.

In this study, we fill an important gap by reporting high-resolution cryo-electron microscopy (cryo-EM) structures of the human ED spliceosome in four consecutive states: two pre-B complexes (mature and late) and two B complexes (early and mature). Discovery of the late ED pre-B complex defies the definition of the pre-B state and reshapes our understanding of the pre-B-to-B transition in vertebrates.^9,11^ Structures of the two B complexes of the ED state reveal insights into spliceosome maturation at the pre-catalytic stage.^10,11^ These advances give rise to a mechanistic model for the assembly of the ED vs ID spliceosome, and reveal insights into canonical splicing, back-splicing, and exon skipping.

## Results

### Preparation and isolation of the ED spliceosomes

The synthetic pre-mRNA contains a central exon bracketed by two partial introns (Fig. 1a; Supplementary information, Fig. S1a). The 5′-intron contains the BPS and 3′-splice site (3′SS); the 3′-intron harbors the 5′SS. This design allows preferential assembly of the ED spliceosomes, but not ID spliceosomes. To facilitate isolation of the ED spliceosomes, pilot experiments were performed to select an exon length (55 nucleotides) that allows back-splicing to proceed slowly in our in vitro assay. Circular exon, a final product of back-splicing by the ED spliceosomes, is detectable after ~120 min (Supplementary information, Fig. S1a).^6,23,27^ Importantly, potential in trans splicing by the spliceosomes, each bound to two pieces of pre-mRNA, is undetectable under the same condition.

Using this in vitro assay, the synthetic pre-mRNA was incubated with the HeLa nuclear extract for 60 min before appearance of circular exon. The 60-min point may help enrich the early-stage ED spliceosomes. The assembled ED spliceosomes were isolated using affinity purification and glycerol gradient centrifugation in the presence of chemical cross-linking reagents (Supplementary information, Fig. S1b). Consistent with our design, the presence of pre-mRNA and all five snRNAs in the sample was confirmed using urea PAGE gel (Supplementary information, Fig. S1c). The purified spliceosomes were examined using negative-staining EM (Supplementary information, Fig. S1d).

### Structures of the human pre-B and B complexes of the ED state

The purified ED spliceosomal complexes were used to prepare cryo-EM samples, which were imaged on a Titan Krio microscope equipped with a Gatan K3 detector. Through processing of 37,699 micrographs, we obtained reconstructions of four distinct ED spliceosomes at average resolutions between 2.6 Å and 3.3 Å (Fig. 1b–g; Supplementary information, Figs. S2–S5 and Tables S1–S3).

For two of the four ED spliceosomes, the overall conformation and protein components are consistent with those of the ID pre-B and ID B complexes.^9–11^ These two spliceosomes are designated mature ED pre-B (3.3 Å, Fig. 1b) and mature ED B (2.7 Å, Fig. 1e). In the mature ED pre-B state (Fig. 1b, g), the RNA helicase PRP28 is well-resolved but U1 snRNP is too flexible to be modeled, likely due to the ATPase activity of PRP28.^18,28^ In contrast, the reconstruction of the mature ED B complex shows fine features, which allowed accurate modeling of the core region, especially for the B-specific factors (Fig. 1e, g).

The third structure of the ED spliceosome closely resembles that of the mature ED B complex but lacks a few B-specific factors. This ED spliceosome is designated early ED B complex (Fig. 1d, g). Notably, the early ED B complex already contains three B-specific factors (CYPH, SMU1 and FBP21) but is yet to recruit at least four additional B-specific factors: UBL5, MFAP1, SNU23, and PRP38, which are present in the mature ED B complex (Fig. 1e, g). As elaborated later, numerous structural features of the early ED B complex set it apart from the mature ED B complex.

The fourth structure reveals a previously unknown state, where the 5′SS already forms a duplex with the ACAGA box of U6 snRNA and the 3′-end sequence of the exon is loaded onto loop I of U5 snRNA (Fig. 1c). Consistent with formation of the U6/5′SS duplex in the ID B state, U1 snRNP is absent and most likely has been released by PRP28. Therefore, the RNA conformation already resembles that of the ID B state. However, both the conformation and location of the protein components in U2 snRNP and tri-snRNP are almost identical to those of the mature ED pre-B state (Fig. 1b, c). For these reasons, this spliceosome is designated the late ED pre-B complex (Fig. 1c). In contrast to the mature ED pre-B state, the catalytic domain of PRP28 is invisible but its N-plug remains attached to tri-snRNP.

U2, U4, U5 and U6 snRNPs in the pre-B or B complex of the ED state adopt the same conformation as those of the ID state.^9–11^ These observations constitute compelling evidence for the notion that, despite different topologies of the pre-mRNA (Fig. 1f), spliceosomes of the ED and ID states likely undergo a similar set of conformational rearrangements during the pre-B-to-B transition.

### Transition from mature ED pre-B to late ED pre-B

**Fig. 2:**
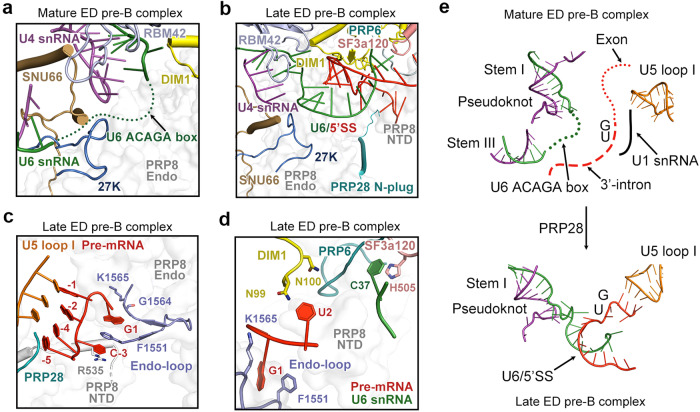
The transition from mature ED pre-B to late ED pre-B. **a** The ACAGA box of U6 snRNA is highly flexible in the mature ED pre-B complex. The local environment for the disordered ACAGA box is shown. **b** The ACAGA box already forms a duplex with 5′SS in the late ED pre-B complex. The surrounding protein components and RNA elements are color-coded. **c** A close-up view on the double-sandwich structure of pre-mRNA recognition in the late ED pre-B complex. The 3′-end sequence of the exon forms a duplex with U5 loop I. The nucleobase of C–3 is flipped out of the duplex registry and sandwiched by Arg535 and Phe1551, both from PRP8. The nucleotide G1 is sandwiched by Phe1551 on one side and Gly1564/Lys1565 on the other side. The bases C–4 and A–5 have weak EM density and are modeled with low occupancies in the final atomic model. **d** A close-up view on the recognition of U6 C37 and 5′SS. His505 of SF3a120 interacts with C37 of U6 snRNA. The Endo-loop grasps G1 of 5′SS; Asn99/Asn100 of DIM1 recognizes U2. **e** Changes of the RNA elements during the transition from mature ED pre-B to late ED pre-B.

The identification of the late ED pre-B complex offers an unprecedented opportunity for understanding the detailed conformational changes in the pre-B-to-B transition. During this transition, 5′SS is released from the U1/5′SS duplex and captured by U6 ACAGA box.^11^ Previously, the ACAGA box is thought to be a free-standing single-stranded RNA in the ID pre-B complex^9^ that forms a duplex with 5′SS in the ID B complex.^11^ In the mature ED pre-B state, the disordered ACAGA box is positioned in the vicinity of four proteins 27K/RBM42/SNU66/DIM1 (Fig. 2a). Quite unexpectedly, the ACAGA box already forms a duplex with 5′SS in the late ED pre-B complex and the N-plug of PRP28 remains associated with PRP8 (Fig. 2b).

During the transition from mature ED pre-B to late pre-B, the endonuclease domain (Endo) and N-terminal domain (NTD) of PRP8, the N-terminal fragment of PRP6, together with four proteins 27K/RBM42/SNU66/DIM1, undergo moderate conformational changes to accommodate the U6/5′SS duplex (Fig. 2a, b). The U6/5′SS duplex in the late ED pre-B complex closely contacts the Endo. The 5′SS is specifically recognized by residues 1548–1566 of the Endo (named the Endo-loop) (Fig. 2c; Supplementary information, Fig. S5a). The nucleotide G1 of 5′SS is sandwiched by Phe1551 and Gly1564/Lys1565 of the Endo-loop, reminiscent of that in the yeast endogenous tri-snRNP or B complex or the human ID B complex.^11,29^ The adjacent nucleotide U2 is stabilized by Asn99/Asn100 of DIM1 (Fig. 2d; Supplementary information, Fig. S5b). In addition, the conformation of the bulged nucleotide C37 of the ACAGA box is stabilized by His505 of SF3a120 and the N-terminal loop of PRP6 through direct interactions (Fig. 2d).

Recognition of the exon sequences is also achieved by loop I of U5 snRNA, which forms a duplex with four nucleotides (G–1, A–2, C–4, and A–5) at the 3′-end of the exon through non-Watson-Crick base pairing (Fig. 2c). Notably, the central base C–3, which is flipped out of the duplex registry, is sandwiched by Arg535 in the NTD and Phe1551 in the Endo-loop of PRP8. The double-sandwich arrangement for G1 and C–3 may greatly strengthen exon recognition. Importantly, in the ED pre-B state, the N-plug of PRP28 appears to have no negative impact on exon recognition by loop I; this structural observation may contrast the proposed function of the N-plug in mimicking the exon and blocking its entry into loop I.^9^ Nonetheless, the N-plug of PRP28 could impede the access of other splicing factors and the complete entry of pre-mRNA in the subsequent B state.

Taken together, the core protein components remain largely unchanged during the mature to late ED pre-B transition, but the RNA elements have undergone pronounced rearrangement (Fig. 2e). U1 snRNA is released, and stem III of the U4/U6 duplex is unwound. 5′SS and the preceding exon are delivered into the spliceosome, forming the U6/5′SS and U5 loop I/exon duplexes in the late ED pre-B complex.

### Transition from late ED pre-B to early ED B

**Fig. 3:**
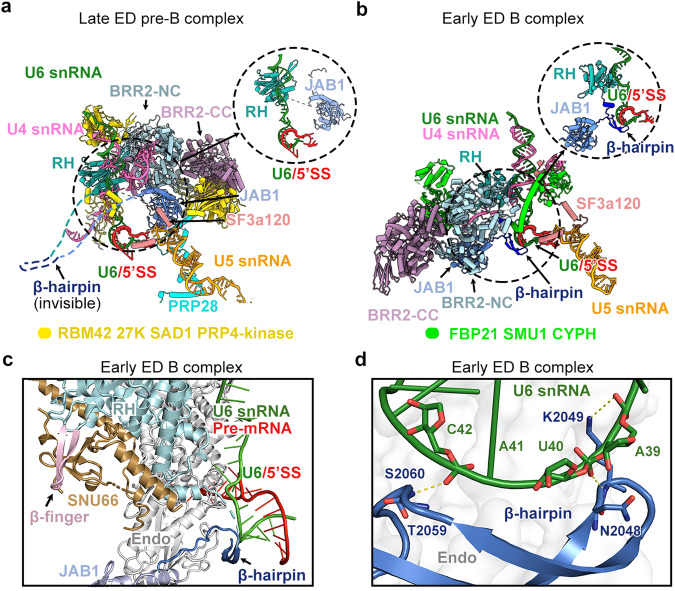
The transition from late ED pre-B to early ED B. **a** Structural features of the late ED pre-B complex. The PRP8 β-hairpin, located between the RH and Jab1 domains, is highly flexible and disordered. The proteins in the core region are color-coded. **b** Structural features of the early ED B complex. The unambiguously assigned PRP8 β-hairpin interacts with the U6/5′SS duplex. U4 snRNA is loaded into the NC of BRR2. **c** A close-up view on the interface between SNU66 and surrounding proteins in the early ED B complex. Residues 248–358 of SNU66 closely interact with the β-finger of the RH domain. **d** A close-up view on the interface between the PRP8 β-hairpin and the U6/5′SS duplex in the early ED B complex. Thr2059 and Ser2060 of the β-hairpin interact with C42 of U6 snRNA. Asn2048 and Lys2049 at the tip of the β-hairpin form H-bonds with the phosphate groups of U40 and A39 of U6 snRNA, respectively.

During the transition of late ED pre-B to early ED B, the pre-B-specific factors, including RBM42, 27K, SAD1, PRP4 kinase and PRP28, are released (Fig. 3a, b). These changes are similar to those of the pre-B-to-B transition observed in the ID spliceosomes.^9,11^ In addition, three B-specific factors (CYPH, FBP21 and SMU1) are recruited into the early ED B complex (Fig. 3b). Dramatic conformational changes occur to the BRR2 helicase; U4 snRNA, just released from U4/U6 stem III, is loaded into its N-terminal cassette (NC) (Fig. 3b).

Notably, a β-hairpin of PRP8, comprising two anti-parallel β-strands, plays a key role in the early ED B complex. The β-hairpin, comprising residues 2040–2060, connects the RNaseH-like domain (RH) with the Jab1 domain of PRP8. These sequences are highly flexible and disordered in the ID pre-B state^9,11^ (Fig. 3a), but adopt a specific conformation and can be unambiguously assigned in the early ED B complex (Fig. 3b; Supplementary information, Fig. S6). During the transition, BRR2 undergoes marked rearrangement, and the accompanying Jab1 domain moves towards the U6/5′SS region (Fig. 3a, b). The RH domain is flipped upside-down, allowing its β-finger to contact a previously uncharacterized region (residues 248–358) of SNU66 (Fig. 3c; Supplementary information, Fig. S5c). This region of SNU66 also binds the Endo domain, thus stabilizing the positional changes of the RH domain. These changes deliver the PRP8 β-hairpin into the vicinity of the U6/5′SS duplex (Fig. 3b, c; Supplementary information, Fig. S6a, b).

The metazoan-specific β-hairpin of PRP8, absent in *Saccharomyces cerevisiae* and *Schizosaccharomyces pombe* (Supplementary information, Fig. S6c), holds the U6/5′SS duplex mainly through interaction with U6 snRNA (Fig. 3d; Supplementary information, Fig. S5d). Thr2059 and Ser2060 of the β-hairpin contact the phosphate backbone of C42 of U6 snRNA. Asn2048 and Lys2049 at the tip of the β-hairpin form hydrogen bonds (H-bonds) with the phosphate groups of U40 and A39 of U6 snRNA (Fig. 3d). Such arrangement suggests a role for the PRP8 β-hairpin in spliceosomal assembly and activation.

### Transition from early ED B to mature ED B

Structures of the human ID B complex have been elucidated at resolutions of 4.5 Å and 3.8 Å.^10,11^ A number of B-specific factors are recruited into the ID B complex.^10,11^ But the moderate resolutions of the ID B complex restrict atomic modeling of the B-specific factors and other spliceosomal components in the core region. These restrictions are lifted, at least partially, by the high-resolution structures of the early and mature ED B complexes.

**Fig. 4:**
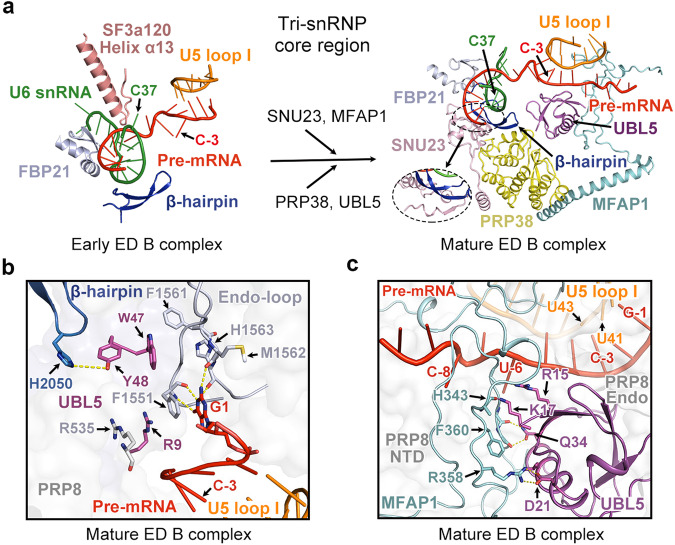
The transition from early ED B to mature ED B. **a** Structural changes around the U6/5′SS duplex during the early ED B to mature ED B transition. The PRP8 β-hairpin, together with FBP21 and SF3a120, interacts with the U6/5′SS duplex in the early ED B complex. Recruitment of SNU23, MFAP1, PRP38, and UBL5 leads to formation of the mature ED B complex. **b** A close-up view on the recognition of the junction region between the exon and 5′SS in the mature ED B complex. Arg9 of UBL5 takes the place of nucleotide C–3 in the late ED pre-B and early ED B states. Trp47 and Tyr48 of UBL5 interact with the Endo-loop and β-hairpin of PRP8, respectively. **c** A close-up view on the tripartite interface among the exon, MFAP1, and UBL5 in the mature ED B complex. MFAP1 and UBL5 interact with each other through an extensive interface. Both proteins stabilize the 3′-end sequences of the exon.

The early B-specific factors that are recruited during the late ED pre-B to early ED B transition include FBP21, CYPH, and SMU1 (Fig. 1g). The major difference between the early and mature ED B complexes is recruitment of four additional B-specific factors to the tri-snRNP: SNU23, PRP38, MFAP1 and UBL5 (Figs. 1 and 4a). Sequential recruitment and detailed arrangement of these B-specific factors are previously unrecognized.

In the late ED pre-B and early ED B states, the nucleotide C–3 from 3′-end of the exon is flipped out of the U5/exon duplex registry (Figs. 2c and 4a, left panel). In the mature ED B state, however, the nucleobase of C–3 is flipped back and pairs up with U5 loop I (Fig. 4a, right panel). Instead, the side chain of Arg9 of UBL5 occupies the location vacated by C–3 and maintains the local conformation involving G1 of 5′SS, Arg535 and Phe1551 of Prp8 (Fig. 4b; Supplementary information, Fig. S5e, f). The β-hairpin of PRP8, which first appears in the early ED B state, forms a β-sheet with an additional β-strand of SNU23 in the mature ED B state (Fig. 4a, right panel). Helix α13 of SF3a120, which contacts the U6/5′SS duplex in the late ED pre-B and early ED B states, is dissociated in the mature ED B state (Fig. 4a). Notably, C37 of U6 snRNA is flipped back into the U6/5′SS duplex, likely due to loss of recognition by helix α13 and PRP6 during the transition (Figs. 2d and 4a).

UBL5 is accommodated in a cavity formed by MFAP1, PRP38, and the NTD and Endo of PRP8 through extensive intermolecular interactions in the mature ED B complex (Fig. 4a, right panel, c). Trp47 of UBL5 directly contacts the Endo-loop; Tyr48 forms an H-bond with His2050 on the tip of the PRP8 β-hairpin (Fig. 4b). These two residues likely contribute to displacement of the nucleotide C–3 by Arg9 of UBL5. Asp21 of UBL5 accepts two charge-stabilized H-bonds from Arg358 of MFAP1 (Fig. 4c). These features are consistent with the observation that UBL5 ablation causes cell death as a result of dysfunction in 5′SS selection for specific introns.^30,31^

A large portion of MFAP1, which remains either unassigned or erroneously assigned as a small fragment from SNU66 in the ID B complex,^10,11^ is unambiguously located (Fig. 4c; Supplementary information, Fig. S5g). MFAP1 closely interacts with nucleotides C–5 through C–9 of the exon, whereas these nucleotides are also in close contact with the NTD of PRP8. By engaging the nucleotides upstream of the exon 3′-end, MFAP1 may stabilize the interaction between the exon and U5 loop I. Together, MFAP1 and UBL5 appear to stabilize the local conformation of the junction region between the exon and 5′SS in the mature ED B complex.

Intriguingly, the EM density for the exon is relatively weak in the late ED pre-B complex (Supplementary information, Fig. S5h) and becomes increasingly stronger in the early ED B complex (Supplementary information, Fig. S5i) and the mature ED B complex after the recruitment of MFAP1 to stabilize the exon (Supplementary information, Fig. S5j). These structural features are consistent with the observed sequential exchange of factors, which allows iterative reinforcement and gradual augmentation of the exon’s interaction with U5 snRNP.

### SF3a120/SF3b145 bridge U2 snRNP and tri-snRNP

**Fig. 5:**
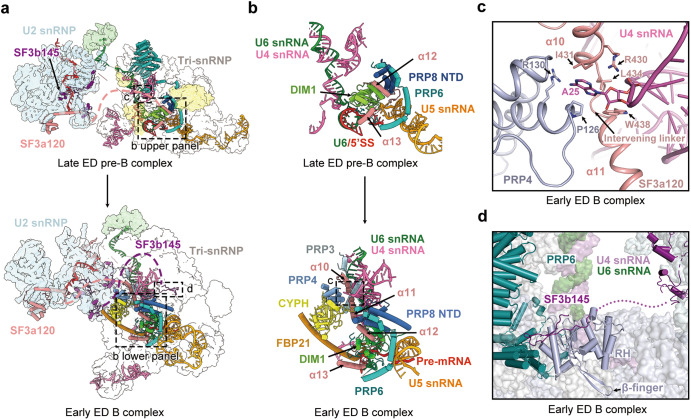
SF3a120 and SF3b145 mediate interactions between U2 snRNP and tri-snRNP in the late ED pre-B and early ED B complexes. **a** The interface between U2 snRNP and tri-snRNP is mediated in part by SF3a120 and SF3b145 in the late ED pre-B complex (upper panel) and early ED B complex (lower panel). SF3a120 and SF3b145 are colored salmon and violet purple, respectively. The disordered regions of these two proteins are indicated by dashed lines. The black dashed boxes, labeled accordingly, highlight the regions that are examined in detail in (**b**–**d**). **b** A close-up view on the interface between U2 snRNP and tri-snRNP in the late ED pre-B complex (upper panel) and early ED B complex (lower panel). Select structural elements of SF3a120 are anchored to the core region of tri-snRNP. The black dashed box highlights the region that is further examined in (**c**). **c** A close-up view on the recognition of the nucleotide A25 from U4 snRNA in the early ED B complex. This nucleotide is stabilized by SF3a120 and the NTD of PRP4. **d** A close-up view on the interface involving the C-terminal fragment (finger loop) of SF3b145 in the early ED B complex. The finger loop contacts the RH domain of PRP8 and the HEAT domains of PRP6 in the early ED and mature ED B complexes.

The interactions between U2 snRNP and tri-snRNP, as observed in the ID pre-B and B complexes at 3.8–4.5 Å resolutions,^9–11^ are mediated by the U2/U6 duplex and SMU1. These features are confirmed by our current structures at 2.6–3.2 Å resolutions. Importantly, our structures of the late ED pre-B and the early and mature ED B complexes unambiguously identify SF3a120 and SF3b145 of U2 snRNP as additional key interactors with the tri-snRNP (Fig. 5; Supplementary information, Figs. S7 and S8). Our structural finding is consistent with the crosslinking-mass spectrometry (MS) results on the human ID pre-B and ID B complexes.^9,10^

In the late ED pre-B complex, conserved helices α12 and α13 of SF3a120 are anchored on the tri-snRNP through direct interactions with PRP6, DIM1, and the NTD of PRP8 (Fig. 5a, b, upper panels; Supplementary information, Fig. S7a). In the early and mature ED B complexes, helices α10 and α11 of SF3a120 further interact with the C-terminal fragments of PRP3, the NTD of PRP4, and CYPH (Fig. 5a, b, lower panels; Supplementary information, Fig. S7b). Notably, helices α10/α11 and the intervening linker recognize a unique “flipped” conformation of the nucleotide A25 of U4 snRNA (Fig. 5c; Supplementary information, Fig. S5k). Specifically, the ribose of A25 is sandwiched by Arg430/Leu434 of α10 on one side and Trp438 of α11 on the other side; the nucleobase of A25 is accommodated in a pocket formed by Ile431 of α10 and Pro126/Arg130 of PRP4 (Fig. 5c; Supplementary information, Fig. S5k). All residues in α10–α13 of SF3a120 that participate in the local interactions are highly conserved from human to yeast (Supplementary information, Fig. S7c).

SF3b145 plays an important role in the early and mature ED B complexes (Fig. 5a, d). An extended sequence at the C-terminus of SF3b145 (residues 781–811), designated as the finger loop (Supplementary information, Fig. S8a, b), has been unambiguously identified to replace the erroneously assigned PRP6 fragment as reported in the ID B complex.^10,11^ The SF3b145 finger loop closely contact the RH domain of PRP8; the tip of the finger loop interacts with the HEAT domains of PRP6 (Fig. 5d; Supplementary information, Fig. S8a). Notably, the location of the finger loop in the early and mature ED B complexes is occupied by the β-finger of the RH domain in the ED pre-B state (Supplementary information, Fig. S8c). During the pre-B-to-B transition, the RH domain is flipped by ~180°, exposing the binding site to the finger loop (Supplementary information, Fig. S8c). SF3b145 residues at these interfaces are highly conserved in higher eukaryotes (Supplementary information, Fig. S8d).

### A working model of the ED pre-B to ED B conversion

**Fig. 6:**
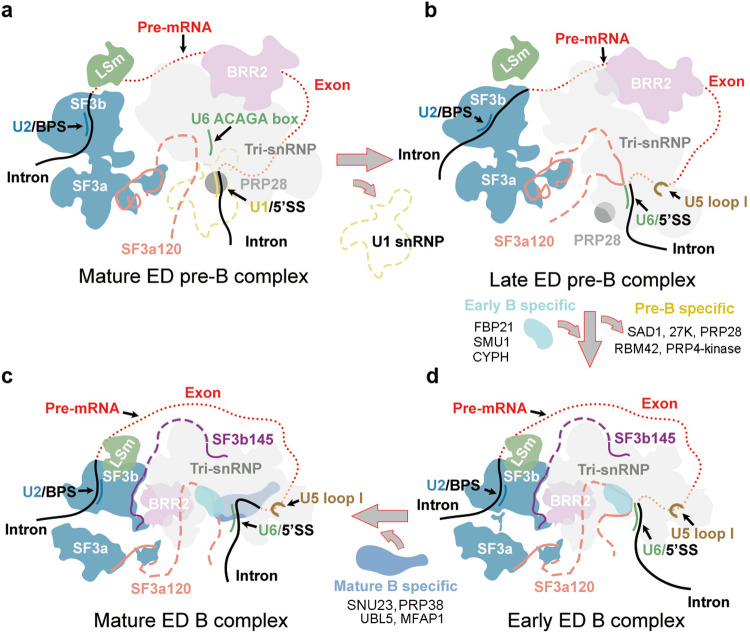
A working model of the spliceosomal remodeling from the mature ED pre-B complex to mature ED B complex. **a** A cartoon diagram of the mature ED pre-B complex. The 5′SS forms a duplex with U1 snRNA. The RNA helicase PRP28 is poised for action. **b** A cartoon diagram of the late ED pre-B complex. Due to the RNA helicase PRP28, the U1/5′SS duplex is unwound and U1 snRNP has been released. The 5′SS already forms a duplex with U6 snRNA and the 3′-end region of the exon is recognized by U5 loop I. Compared to the mature ED pre-B complex, the protein components in the core region remain largely unchanged. **c** A cartoon diagram of the early ED B complex. The pre-B-specific factors have been released. Three early B-specific factors (FBP21, SMU1 and CYPH) have been recruited. Compared to the late ED pre-B complex, a number of protein components exemplified by BRR2 display marked conformational changes. **d** A cartoon diagram of the mature ED B complex. Four additional mature B-specific factors (SNU23, MFAP1, PRP38 and UBL5) have been recruited into the tri-snRNP.

Based on our structural advances, we propose a three-step model for the conversion from mature ED pre-B complex to mature ED B complex (Fig. 6). During the first step from mature ED pre-B to late ED pre-B, 5′SS is released by PRP28 from the U1/5′SS duplex and captured by U6 ACAGA box, and U5 loop I recognizes the exon 3′-region (Fig. 6a, b). SF3a120 enters the tri-snRNP and interacts with the U6/5′SS duplex. In contrast to the RNA changes, the composition and conformation of the core protein components remain quite similar between these two states.

Major changes of the protein components occur in the second step from late ED pre-B to early ED B (Fig. 6b, c). A number of pre-B-specific factors are released and three early B-specific factors are recruited to the early ED B complex. BRR2 is translocated to its functional position and U4 snRNA is loaded into its NC (Fig. 3a, b). The β-hairpin of PRP8, formed in the early ED B state, stabilizes the U6/5′SS duplex by interacting with U6 snRNA (Fig. 3d). Helices α10 and α11 of SF3a120 that are loaded onto the U4/U6 duplex may facilitate recruitment of PRP4 NTD and the B-specific factors such as CYPH and FBP21 (Fig. 5b, c).

During the third step from early ED B to mature ED B, four mature B-specific factors are recruited to the vicinity of the U6/5′SS duplex (Fig. 6c, d). SNU23 interacts with the β-hairpin of PRP8 and may further stabilize the U6/5′SS duplex (Fig. 4a). Together with the Endo-loop, UBL5 stabilizes the recognition of 5′SS G1, contributing to exon binding. Due to competition with Arg9 of UBL5, C–3 of the exon, which is out of duplex registry in the late ED pre-B and early ED B complexes, is flipped back and base-pairs with U5 loop I in the mature ED B complex (Fig. 4b, c). MFAP1 also contributes to recognition of the exon 3′-region and stabilizes the exon/U5 loop I duplex.

## Discussion

Base flipping of C–3 observed in the late ED pre-B and early ED B complexes is likely a general feature in human RNA splicing. The base C–3 forms a double-sandwich with G1 of the intron and conserved PRP8 residues; these interactions may stabilize the U5 loop I/exon duplex (Fig. 2c). The C–3 position of the double-sandwich structure is predicted to accommodate not just the base cytosine, but also the other three bases. Influenced by the local conformation, the nucleotide A–2 within the exon of the late ED pre-B or the early and mature ED B complex exists in the *syn* configuration, which differs from the *anti* configuration as observed in the activated spliceosome.^12,32–36^ In the mature ED B complex and beyond (such as B^act^), the C–3 base is flipped back into registry (Fig. 4b); the U5 loop I/exon duplex is in turn stabilized by other factors, which include UBL5 in the mature ED B complex and SRm300 and the PRP8 switching loop in the ID B^act^ through P complexes (Supplementary information, Fig. S9).

**Fig. 7:**
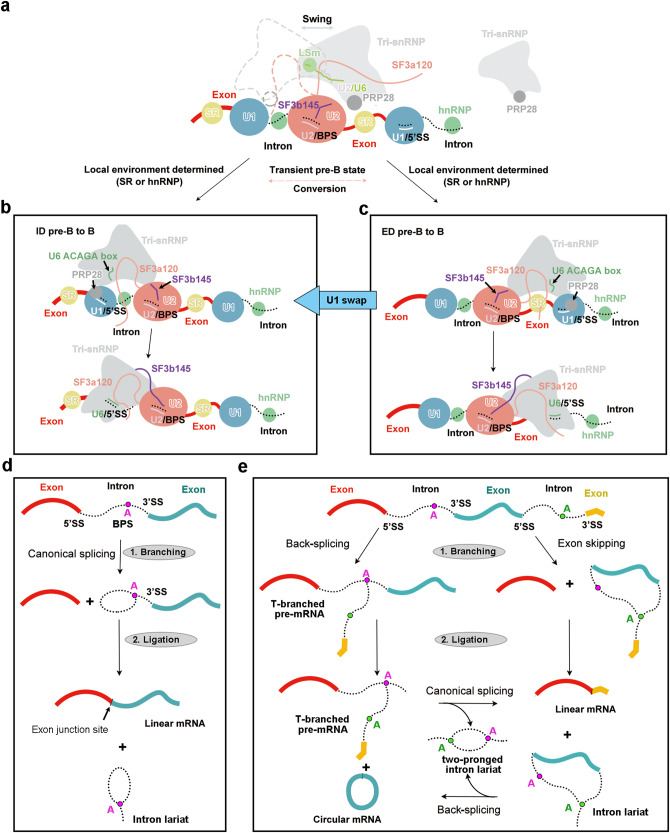
A comprehensive model on canonical splicing, back-splicing, and interconversion between the ID and ED spliceosomes. **a** A carton diagram of the transient pre-B complex assembled on an endogenous transcript. U2 snRNP is assembled on the central intron, upstream of the central exon. One U1 snRNP is assembled on the central intron, whereas another U1 snRNP is located downstream of the central exon. Free tri-snRNP with PRP28 may be recruited by SF3a120 and U2 snRNA.^18^ Depending on which U1 snRNP it engages, the SF3a120-linked tri-snRNP may assemble into an ID or ED pre-B complex. **b** Assembly of the ID pre-B complex and its conversion to the ID B complex. With the tri-snRNP engaging U1 snRNP on the central intron, the ID pre-B complex is assembled. PRP28 unwinds the U1/5′SS duplex, leading to U1 snRNP release and U6/5′SS duplex formation in the B complex. **c** Assembly of the ED pre-B complex and its conversion to the ED B complex. Engagement of the tri-snRNP with U1 snRNP downstream of the central exon results in assembly of the ED pre-B complex, which may be converted to the ED B complex by PRP28. But the ED pre-B complex may also be converted to the ID pre-B complex if the tri-snRNP switches over to engage the U1 snRNP on the central intron. **d** A schematic diagram of canonical splicing as mediated by the ID spliceosomes. **e** A schematic diagram of back-splicing as mediated by the ED spliceosomes. Circular exon is a final product of back-splicing by ED spliceosomes. Several scenarios are shown here.

In the human genome, the average length of introns vastly exceeds that of exons. Therefore, an early-state spliceosome is more likely to be assembled across an exon than an intron. Such an ED spliceosome must be regularly converted to the ID state for mRNA to be produced. In the early A complex, U1 snRNP already recognizes pre-mRNA through the U1/5′SS duplex but U2 snRNP is yet to engage the BPS (Supplementary information, Fig. S10a). The interaction between the UBL domain of SF3a120 and the SL4 of U1 snRNA^5,37–39^ may facilitate U2 snRNP to recognize the BPS of the upstream intron (Supplementary information, Fig. S10b). This analysis provides a plausible explanation to reduced upstream loading of U2 snRNP in response to obstruction of U1 snRNP binding to downstream 5′SS.^5^ It also explains the enhanced association of U2 snRNP with the single exon RNA in the presence of U1 snRNP.^4^ Notably, formation of the A complex leads to abrogation of the interaction between UBL and the downstream U1 snRNA (Supplementary information, Fig. S10b). U2 snRNP then recruits the tri-snRNP through U2 snRNA and the flanking sequences of SF3a120 (Supplementary information, Fig. S10c), forming a proposed transient pre-B complex (Fig. 7a).

In the transient pre-B complex, the floating tri-snRNP may bind the U1/5′SS duplex on either side of U2 snRNP, resulting in formation of the ID or ED pre-B complex (Fig. 7b, c).^9,11,18,40^ This process may be regulated by exon-dependent SR proteins or intron-dependent hnRNP proteins.^24–26,41,42^ In the ID pre-B complex, PRP28 unwinds the U1/5′SS duplex, allowing formation of the U6/5′SS duplex and consequent conversion of the ID pre-B complex to the ID B complex (Fig. 7b). Similarly, PRP28 converts the ED pre-B complex to the ED B complex (Fig. 7c). In the case of pre-mRNA co-transcriptional splicing by the ID spliceosomes, splicing of an upstream intron is presumably ahead of its adjacent downstream intron; this is confirmed by human genome-wide sequencing analysis.^43,44^

Although an intron may contain thousands of nucleotides, these sequences may have defined folding patterns that are facilitated by specific hnRNP proteins.^45^ Consequently, the 5′SS and 3′SS of the same intron may not be separated far away from each other. This feature overcomes the distance obstacle for long introns and facilitates the upstream engagement process. In this regard, splicing of specific sequences by the ID spliceosomes is already encoded by the genome sequences.^43,44^ Endogenetic transcripts mainly undergo splicing by the ID spliceosomes (Fig. 7d).

The transient pre-B state may act as a critical juncture for the spliceosome to determine whether splicing will proceed along the ID or ED pathway. Importantly, however, the ID-vs-ED conversion may occur at the pre-B state (Fig. 7b, c, indicated by a thick arrow). This conversion is likely driven by U1 swapping: the tri-snRNP dissociates from the downstream U1 snRNP and associates with the upstream U1 snRNP in the case of ED-to-ID conversion. Once the B complex is formed, the ED-to-ID conversion becomes highly unlikely due to the numerous interactions between tri-snRNP and pre-mRNA including the U6/5′SS and U5/5′-exon duplexes and many associated proteins. At the B state, the ED-to-ID conversion literally entails disassembly of the entire spliceosomal complex. Notably, there was no differentiation between the pre-B and B states until about a few years ago^18^; therefore, the B-like complex discussed in the ED-to-ID conversion prior to this time likely represented the pre-B complex.^5^

Compared to canonical splicing by the ID spliceosome (Fig. 7d), splicing by the ED spliceosomes generates more possibilities (Fig. 7e). On one hand, back-splicing results in a characteristic T-branched RNA intermediate that further produces a circular exon and a shorter T-branched RNA. The latter may go through canonical splicing to yield a ligated exon and a two-pronged intron lariat (Fig. 7e). On the other hand, a stable ED spliceosome may lead to exon skipping and allow generation of an exon-containing lariat RNA, which then undergoes back-splicing to produce a circular exon and a two-pronged intron lariat (Fig. 7e). These scenarios merely serve as examples of the far more complex situation in cells. The circular exon, generated by the ED spliceosomes,^6,23,27^ is detectable in our assay system (Supplementary information, Fig. S1a). Our analysis is consistent with the observation that exon skipping appears to be correlated with exon circularization.^46,47^ The skipped circular exons can be detected by RT-PCR, but not by genome-wide RNA analysis.^47^ This suggests the low efficiency of back-splicing in cells, which is consistent with appearance of circular RNA only after prolonged reaction time in our in vitro splicing system (Supplementary information, Fig. S1a).

An important finding of our study is structural elucidation of the late ED pre-B complex, which is likely a general feature of both ED and ID spliceosomes in higher organisms. This structure sheds light on the key question of how the 5′SS and 5′-exon are delivered into U6 snRNA and U5 loop I, respectively. It also reveals the essential structural rearrangements that precede BRR2 translocation and may explain the perplexing question of why the human pre-B and B complexes are very different but their yeast counterparts are quite similar. In the yeast ID pre-B and ID B complexes, U5 loop I is constantly occupied by U6 snRNA (Supplementary information, Fig. S11a, b). The replacement of U6 snRNA by 5′-exon-5′SS is a prerequisite for yeast spliceosome activation. In contrast, U5 loop I is free of U6 snRNA or pre-mRNA in the human ID pre-B complex (Supplementary information, Fig. S11c) but already engages the 5′-exon-5′SS through duplex formation in the human ID B complex (Supplementary information, Fig. S11d). Our structure of the human late ED pre-B complex shows that this process occurs before any major changes of the protein components (Supplementary information, Fig. S11e, f). The subsequent protein changes result in the ED B complex (Supplementary information, Fig. S11g, h). Therefore, the large differences between the human pre-B and B complexes are likely necessitated by the ED-to-ID transition. In the budding yeast, however, there is no ED spliceosome and hence no such ED-to-ID transition.

The structure of the human late ED pre-B complex may also explain why this process is less efficient than canonical splicing by the ID spliceosomes. In the ED pre-B complex, the downstream 5′SS and upstream BPS/3′SS are connected by an exon, designated as the ED path (Supplementary information, Fig. S12a, b). During the pre-B-to-B transition, the exon within the ED pre-B complex may physically interfere with the translocation of the BRR2 helicase (a large, 245-kDa protein), because BRR2 must push away, or be translocated underneath, the exon RNA strand. This process likely results in prolonged dwelling time of the ED spliceosome in the pre-B state, especially for short exons. In contrast, for the transition of ID pre-B to ID B (Supplementary information, Fig. S12c, d), BRR2 does not touch, or translocate underneath, the intron and is thus unobstructed by the intron. The distance between the 5′-end of 5′SS and the 3′-end of 3′SS is ~250 Å in the human pre-B complex. Taking into account the physical attributes of the spliceosome, the exon must span a distance of ~300 Å, which necessitates an RNA sequence of ~50 nucleotides. An exon shorter than this length may not provide sufficient space for efficient translocation of BRR2. Consistent with this analysis, the length of circular RNA mostly exceeds 100 nucleotides. Our analysis of a human circRNA database, comprising 4635 circular RNAs, reveals that the majority fall within the range of 150 to 500 nucleotides (Supplementary information, Fig. S13a). The length distribution is consistent with our structural observations.

In this study, prolonged incubation of the synthetic pre-mRNA with nuclear extract allowed assembly and purification of the ED spliceosomes in the absence of any splicing inhibitor or modulator. Remarkably, these ED spliceosomes are mostly in the pre-B and B states. This outcome is achieved after careful pilot experiments to determine the length of the exon. These observations suggest two kinetic barriers: the remodeling of the ED pre-B complex and the activation of the ED B complex. The relatively stable late ED pre-B complex may pose an obstacle for conformational changes. This obstacle could be the RNA path imposed by the relatively short exon, which restricts BRR2 translocation as required by the ED pre-B to ED B transition. Such restriction is unique to the ED, but not the ID, spliceosomes.

The average resolution for the reconstructions of the ED spliceosomes is improved over that of their ID counterparts.^11^ This is likely due to the design of the pre-mRNA, which in this study contains a single exon of 55 nucleotides in the center and two flanking half introns. Such a design allows preferential accumulation of the early ED spliceosomes (pre-B and B) and may reduce conformational flexibility of these ED spliceosomes, both contributing to improved resolution.

The short exon not only connects U2 snRNP with tri-snRNP but also serves as the binding site for SR proteins. These circumstances may create a constraint on the movement of U2 snRNP, thus slowing down the activation of the ED B complex. In contrast, U2 snRNP movement is unrestricted in the ID B complex. In addition, the short exon in the ED B complex may reinforce the interaction between U2 snRNP and tri-snRNP, making the ED B complex more stable compared to the ID B complex (Supplementary information, Fig. S13b, lower ED path). In the case the short exon in the ED pre-B state moves along with BRR2 or just locates in the ID path, the resulting ED B complex will have a topological barrier that may interfere with the conformational transition of BRR2 to the B^act^ structure (Supplementary information, Fig. S13b, upper ED path). All these considerations may slow down the action of the RNA helicase BRR2.

These analyses also shed light on how the ED-to-ID transition may occur for human spliceosomes (Supplementary information, Fig. S14). Following the formation of the human late ED pre-B complex, the exon locks the pre-B complex in place for a prolonged period. During this time, the upstream U1 snRNP has a higher likelihood to be recruited into the ED complex, allowing the upstream 5′SS and 5′-exon to compete and replace the downstream 5′SS and 5′-exon, which have been paired up with U5 loop I in a loosely assembled spliceosome. This process results in the conversion of the ED pre-B complex into the ID pre-B complex (Supplementary information, Fig. S14).

Our pre-mRNA only allows initial assembly of the ED pre-B complex, not ID pre-B complex. The ED-to-ID conversion in trans is only possible when an additional piece of pre-mRNA is involved. In this case, U1 snRNP of the ED pre-B complex together with the bound 5′SS must first dissociate from the tri-snRNP; then the tri-snRNP must recruit another U1 snRNP that is bound to another pre-mRNA. This process is thermodynamically unfavorable, because it involves disruption of intramolecular interaction and formation of intermolecular interaction. This process is quite different from the ED-to-ID transition discussed in this study. In fact, *trans* splicing is rare in humans.^48^

## Materials and methods

### Cell lines

Hela S3 cell lines were cultured in SMM 293 TI medium without FBS at 37 °C in 5% CO_2_ and used to prepare the nuclear extract.

### In vitro splicing assay

The pre-mRNA used in the experiment was m^7^G (5′) ppp (5′)-capped and in vitro synthesized using the T7 runoff transcription method. The RNA sequence was derived from the adenovirus. The nuclear extract was prepared from HeLa S3 cells as described.^49^ A typical splicing reaction was performed at 30 °C with 20 nM pre-mRNA and 40% HeLa nuclear extract in a reaction buffer containing 3 mM MgCl_2_, 65 mM KCl, 20 mM HEPES-KOH pH 7.9, 2 mM ATP and 20 mM creatine phosphate.

### Design of the pre-mRNA sequence for ED spliceosome assembly

The pre-mRNA construct used for the human spliceosome purification consists of an exon in the middle and two partial introns on the 5′- and 3′-ends of the exon (Supplementary information, Fig. S1a). This design allows preferential assembly of the ED spliceosomes, but not ID spliceosomes. To facilitate isolation of the ED spliceosomes, we performed pilot experiments to determine the length of the exon length that allows back-splicing to proceed slowly in our in vitro assay. In the end, we chose 55 nucleotides, which allow detection of the circular exon, a final product of back-splicing by the ED spliceosomes, after ~120 min in our in vitro splicing assay. Notably, under these same conditions, we have never been able to detect the ligation of two exons, a predicted *trans*-splicing product of the spliceosome bound to two pieces of pre-mRNA.

The final pre-mRNA contains 144 nucleotides (nt). The 55-nt central exon has the sequence 5′-GGGCGAAUUCGAGCUCACUCUCUUCCGCAUCGCUGUCUGCGAGGUACCCUACCAG-3′. The 52-nt upstream partial intron is 5′-GGGAGGUUUCCUUGAAGCUUUCGUGCUG**A**CCCUGUCCCUUUUUUUUCCAC**AG**-3′, and the 37-nt downstream partial intron has the sequence 5′-**GU**GAGUAUGGAUCCCUCUAAAAGCGGGCAUGACUUCU-3′. The upstream intron contains the BPS and the poly-U tract, and the downstream intron contains the 5′SS.

### Purification of the human ED spliceosomal complexes

A triple MS2-binding site was placed at the 3′-end of the pre-mRNA construct. The MS2-MBP protein was pre-incubated with pre-mRNA before the splicing reaction. The splicing reaction was incubated at 30 °C for 1 h, and then MS2-MBP affinity purification was performed (Supplementary information, Fig. S1b). The G150-4% buffer used for sample washing contains 20 mM HEPES-KOH, 150 mM NaCl, 1.5 mM MgCl_2_, and 4% glycerol. Finally, the complex was eluted using the G150-4% buffer supplemented with 20 mM maltose.

### Gra-Fix crosslinking

The elution after MS2-MBP affinity purification was applied to a 38.2 mL 10%–30% linear glycerol gradient in the G150 buffer supplemented with 0%–0.2% EM-grade glutaraldehyde.^50^ The sample was fractioned every 2 mL from top to bottom after 13.5-h centrifugation at 25,300 rpm using an SW32 rotor (Beckman Coulter) at 4 °C. The RNA components in the sample were analyzed on 8% denaturing polyacrylamide gels (Supplementary information, Fig. S1c). Fractions that contain the spliceosomal complexes were pooled and concentrated to a final volume of ~2 mL. Glycerol in the sample was removed through dialysis against the G150 buffer overnight.

### Circular RNA detection

The synthetic pre-mRNA was incubated with HeLa cell nuclear extract in a 20-μL volume to allow in vitro splicing reaction to proceed. The canonical splicing, executed by the ID spliceosomes each bound to two pieces of synthetic pre-mRNA, was completed after ~30 min. The back-splicing, executed by the ED spliceosomes each bound to a single piece of synthetic pre-mRNA, occurred much more slowly. The total RNA was extracted from after 120 min of incubation. One microgram of RNA was digested using 5 units of RNase R (Epicenter) at 37 °C for 1 h. The digested products were directly reverse-transcribed to produce cDNA using the circular-reverse primer: 5′-CGGAAGAGAGTGAGCTCGAA-3′. Then, the transcribed cDNA was further amplified through PCR using the circular-reverse primer and the circular-forward primer: 5′-CATCGCTGTCTGCGAGGTAC-3′. The PCR products were analyzed using 5% high sieving agarose gels (YEASEN) (Supplementary information, Fig. S1a). The band that corresponds to the suspected circular exon was purified from the gel and confirmed through DNA sequencing.

### EM sample preparation

The dialyzed sample was examined using negative staining and used for cryo-EM sample preparation after concentration. For negative staining, 4 μL sample was loaded on the glow-discharged, carbon film-coated copper grid (Zhongjingkeyi Technology Co. Ltd.). After 90-s absorption, the sample was stained using uranyl acetate (2% w/v) and imaged on an FEI Tecnai Spirit Bio TWIN microscope that operated at 120 kV (Supplementary information, Fig. S1d).

Cryo-EM grids were prepared using Vitrobot Mark IV (FEI Company) at 8 °C and 100% humidity. Four-microliter aliquots of the sample were applied to the glow-discharged homemade carbon filmed Au grid (Quantifoil), blotted for 3 s, and plunged into liquid ethane cooled by liquid nitrogen.

### EM data acquisition

The cryo-EM grids were imaged on a Titan Krios microscope operating at 300 kV and equipped with a Gatan K3 detector and a GIF Quantum energy filter (slit width 20 eV) (Supplementary information, Fig. S2a). Micrographs were recorded in the super-resolution mode with a calibrated pixel size of 0.550 Å. Each stack of 32 frames was exposed for 8 s, with a dose rate of about 4.7 e^–^/s/Å^2^. Automated data collection was performed using AutoEMation.^51^ All 32 frames in each stack were aligned and summed using the whole-image motion correction program MotionCor2^52^ and binned to a pixel size of 1.1 Å. The defocus value of each image, which was set from –1.5 to –2.0 μm during data collection, was determined using Gctf.^53^

### Data processing

For the human spliceosomal dataset, 6,936,951 particles were automatically picked using Gautomatch (https://www2.mrc-lmb.cam.ac.uk/download/gautomatch-053/) from 37,699 micrographs.

Low-resolution maps from human ID pre-B and ID B complexes (EMDB codes: EMD-9621 and EMD-9624) and two bad references generated through conventional 3D classifications were used to perform the “guided multi-reference classification”^32^ in cryoSPARC hetero-refinement.^54^ Two runs of guided multi-reference global 3D classification were performed, resulting in a dataset of 1,220,608 or 673,200 particles for the ED B or ED pre-B complexes, respectively. Because the ED pre-B and ED B particles suffered severe orientation bias on the carbon grids as judged by 2D classification (Supplementary information, Fig. S2b), additional particle picking strategy was executed to reduce its negative impact on reconstruction. Using the coordinates of these 1,893,808 ED pre-B/B particles as additional input for Gautomatch, one round of exclusive particle picking was performed, which resulted in 4,889,307 distinct particles (Supplementary information, Fig. S2c). Using a “Seed facilitated 3D classification” strategy,^55^ 1,400,299 and 893,213 particles for the ED pre-B and ED B complexes were generated for further calculation.

After removal of duplicated particles, a total of 2,597,411 particles from the two sets of particles were selected, re-centered, and re-extracted using a pixel size of 1.1 Å in RELION.^56^ After one additional round of guided multi-reference 3D classification followed by local and CTF refinement in CRYOSPARC,^54^ 1,130,369 and 613,462 particles gave rise to EM reconstructions at average resolutions of 2.7 Å and 3.2 Å for the ED B and ED pre-B complexes, respectively. After Bayesian polishing in RELION,^56^ the average resolution for the reconstruction of the ED B complex was improved to 2.4 Å (Supplementary information, Fig. S2c).

The BRR2 region of the ED pre-B complex with a soft mask was further refined using CRYOSPARC,^54^ yielding a reconstruction with an average resolution of 3.2 Å (Supplementary information, Fig. S2c). Additional global 3D classification led to the identification of the mature and late ED pre-B complexes, which were refined to average resolutions of 3.25 Å and 3.2 Å, respectively (Supplementary information, Figs. S2c and S3a, b and Table S1).

For the BRR2 region of the ED B complex, focused 3D classification and refinement with a soft mask were performed using RELION,^56^ resulting in 765,228 particles and a reconstruction with an average resolution of 3.2 Å (Supplementary information, Fig. S2c). Further 3D classification that focused on the regions of mature B-specific factors led to the identification of the early and mature ED B complexes, which were refined to average resolutions of 2.6 Å and 2.7 Å, respectively (Supplementary information, Figs. S2c and S4a, b).

The angular distribution of the particles used for the final reconstruction of the ED pre-B and ED B complexes are reasonable (Supplementary information, Figs. S3a, b and S4a, b). The local resolution for the core regions of the ED pre-B and ED B complexes and the BRR2 region reaches 2.8 Å, 2.3 Å, and 3.0 Å, respectively (Supplementary information, Figs. S3c and S4c). Local resolution variations were estimated using CRYOSPARC.^54^ Reported resolutions were calculated on the basis of the Fourier shell correlation (FSC) 0.143 criterion, and the FSC curves were corrected with high-resolution noise substitution methods.^57^

### Model building and refinement

The atomic coordinates of the pre-B and B complexes of the ED state were generated by combining rigid docking, homology modeling and de novo model building. Cryo-EM structures of the human ID pre-B (PDB codes: 6AH0 and 6QX9) and ID B (PDB code: 6AHD) complexes were used as initial structural models for the ED pre-B and ED B complexes, respectively. The structures were docked into the EM density maps and manually adjusted and re-built using COOT.^58^ Additional sequences of SF3a120 and SF3b145, the PRP8 β-hairpin between the RH and Jab1 domains, UBL5, MFAP1, SNU23, the PRP8 β-finger binding region of SNU66, and pre-mRNA were manually identified and modeled using COOT^58^ (Supplementary information, Table S1). The relatively mobile U6 LSm, U5 Sm and U4 Sm rings and U2 snRNP were rigid-body docked into the EM density with all side chains removed (Supplementary information, Tables S2 and S3).

The atomic models for the core regions of the ED pre-B and ED B complexes were refined against the corresponding maps using PHENIX^59^ in real space with secondary structure and geometry restraints (Supplementary information, Figs. S3d, e and S4d, e). The structures of all four complexes were validated through examination of the Clash scores, Molprobity scores and statistics of the Ramachandran plots in PHENIX^59^ (Supplementary information, Table S1). The EM maps are of good quality (Supplementary information, Figs. S5, S6b, S7a, b and S8b).

### Supplementary information

Supplementary information, Figure S1

Supplementary information, Figure S2

Supplementary information, Figure S3

Supplementary information, Figure S4

Supplementary information, Figure S5

Supplementary information, Figure S6

Supplementary information, Figure S7

Supplementary information, Figure S8

Supplementary information, Figure S9

Supplementary information, Figure S10

Supplementary information, Figure S11

Supplementary information, Figure S12

Supplementary information, Figure S13

Supplementary information, Figure S14

Supplementary information, Table S1

Supplementary information, Table S2

Supplementary information, Table S3

## Data Availability

The atomic coordinates of the mature and late ED pre-B and the early and mature ED B complexes have been deposited in the Protein Data Bank (PDB) with the accession codes 8H6J, 8H6E, 8H6L and 8H6K, respectively. The EM maps have been deposited in the Electron Microscopy Data Bank (EMDB) with the accession codes EMD-34505, EMD-34500, EMD-34508 and EMD-34507. The local EM maps for the BRR2 region of the mature ED pre-B and early ED B complexes have been deposited as additional maps in EMD-34505 and EMD-34508, respectively.
