# Supplementary material for: Structural insights into human exon-defined spliceosome prior to activation
Source: Cell Res. 2024 Apr 24;34(6):428–39. doi: 10.1038/s41422-024-00949-w (PMC11143319; doi:10.1038/s41422-024-00949-w)
Supplement: Supplementary file 1 — Supplementary information, Figure S1 [file 41422_2024_949_MOESM1_ESM.pdf]

Supplementary information

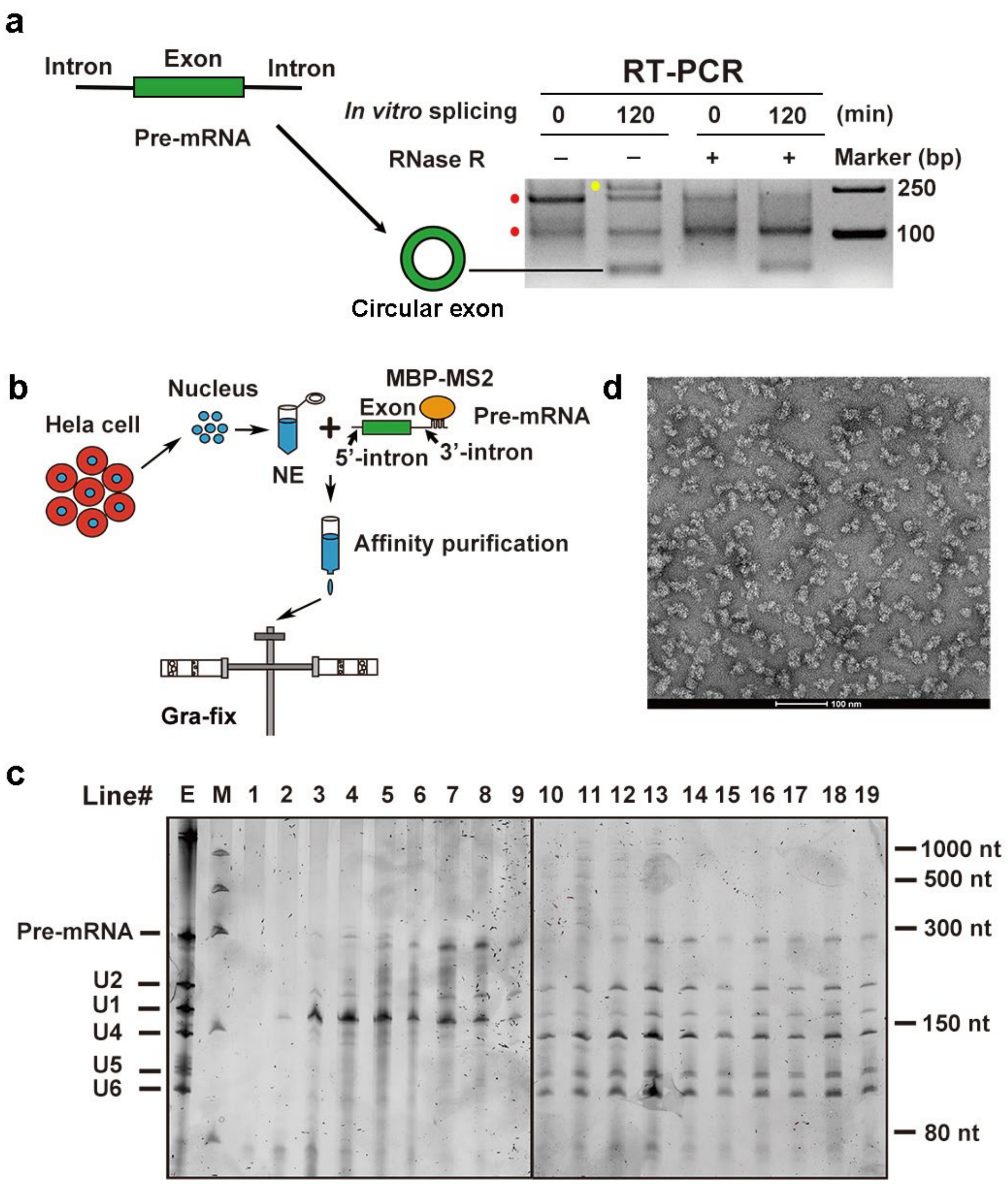

Fig. S1 Purification of human ED spliceosomes for cryo-EM analysis. a Detection of circular exon. The circular exon (in green circle), generated through back-splicing, was detected by reverse transcription-PCR (RT-PCR) after 120 minutes *in vitro* splicing reaction followed by RNase R digestion. The band that represents the circular RNA product was confirmed by DNA sequencing. Other bands on the gel

include contaminated RNA from the NE (red dots) and linear RNA (yellow dot). **b** A schematic diagram of the purification protocol for human ED spliceosomes. **c** Analysis of the RNA components on urea PAGE gels. Various fractions from centrifugation were analyzed for their RNA components. **d** A representative negative staining micrograph of the spliceosome particles after Gra-Fix.
