# Supplementary material for: Structural insights into human exon-defined spliceosome prior to activation
Source: Cell Res. 2024 Apr 24;34(6):428–39. doi: 10.1038/s41422-024-00949-w (PMC11143319; doi:10.1038/s41422-024-00949-w)
Supplement: Supplementary file 2 — Supplementary information, Figure S2 [file 41422_2024_949_MOESM2_ESM.pdf]

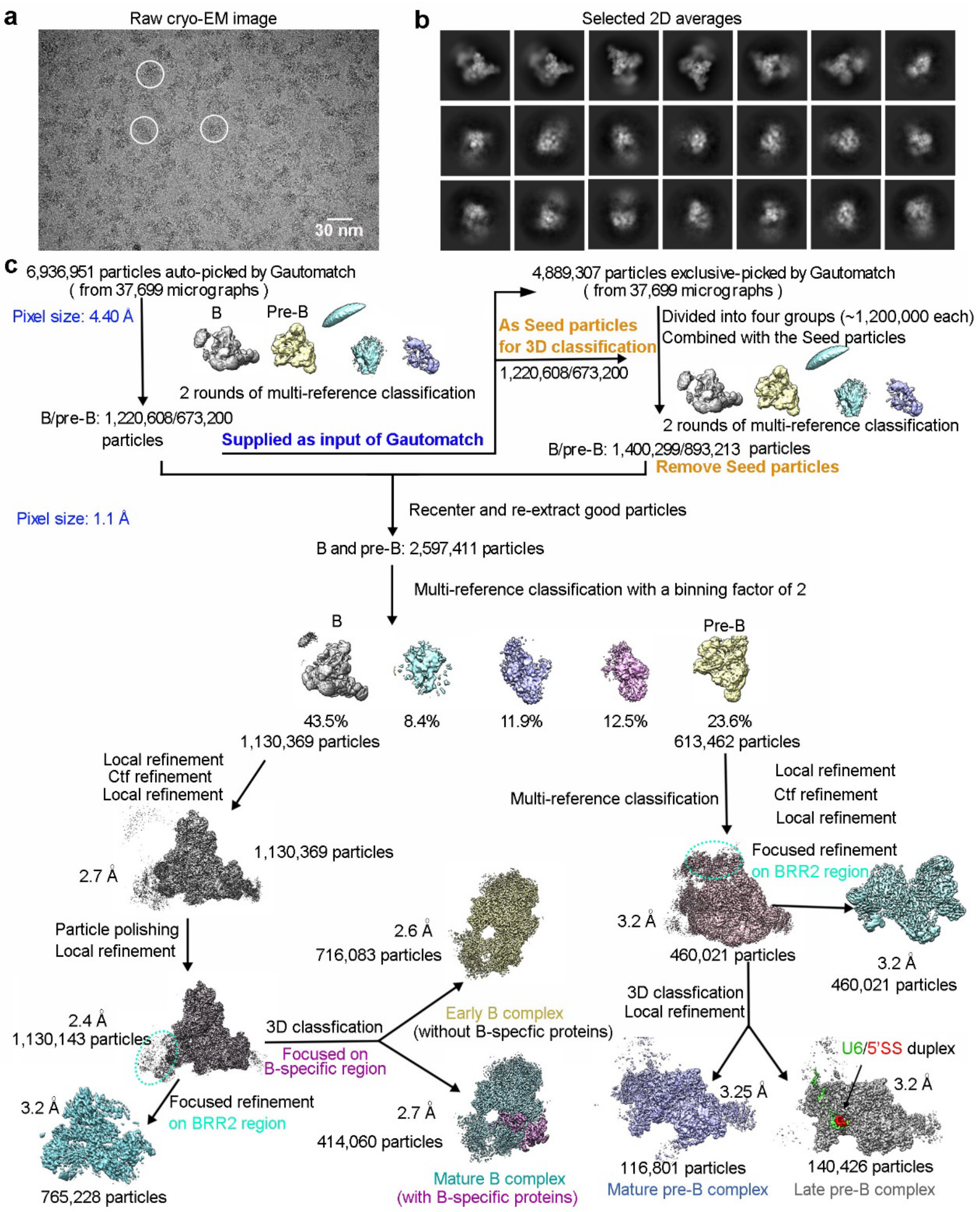

Fig. S2 Data processing workflow of the purified ED spliceosomes. **a** A representative cryo-EM micrograph of the spliceosomal particles. **b** Representative 2D class averages of the spliceosomal particles. **c** Data processing workflow of the ED spliceosomal particles. Details can be found in the Methods. All EM volumes in Fig. S2-S4 were prepared using ChimeraX [60].
