# Supplementary material for: Structural insights into human exon-defined spliceosome prior to activation
Source: Cell Res. 2024 Apr 24;34(6):428–39. doi: 10.1038/s41422-024-00949-w (PMC11143319; doi:10.1038/s41422-024-00949-w)
Supplement: Supplementary file 3 — Supplementary information, Figure S3 [file 41422_2024_949_MOESM3_ESM.pdf]

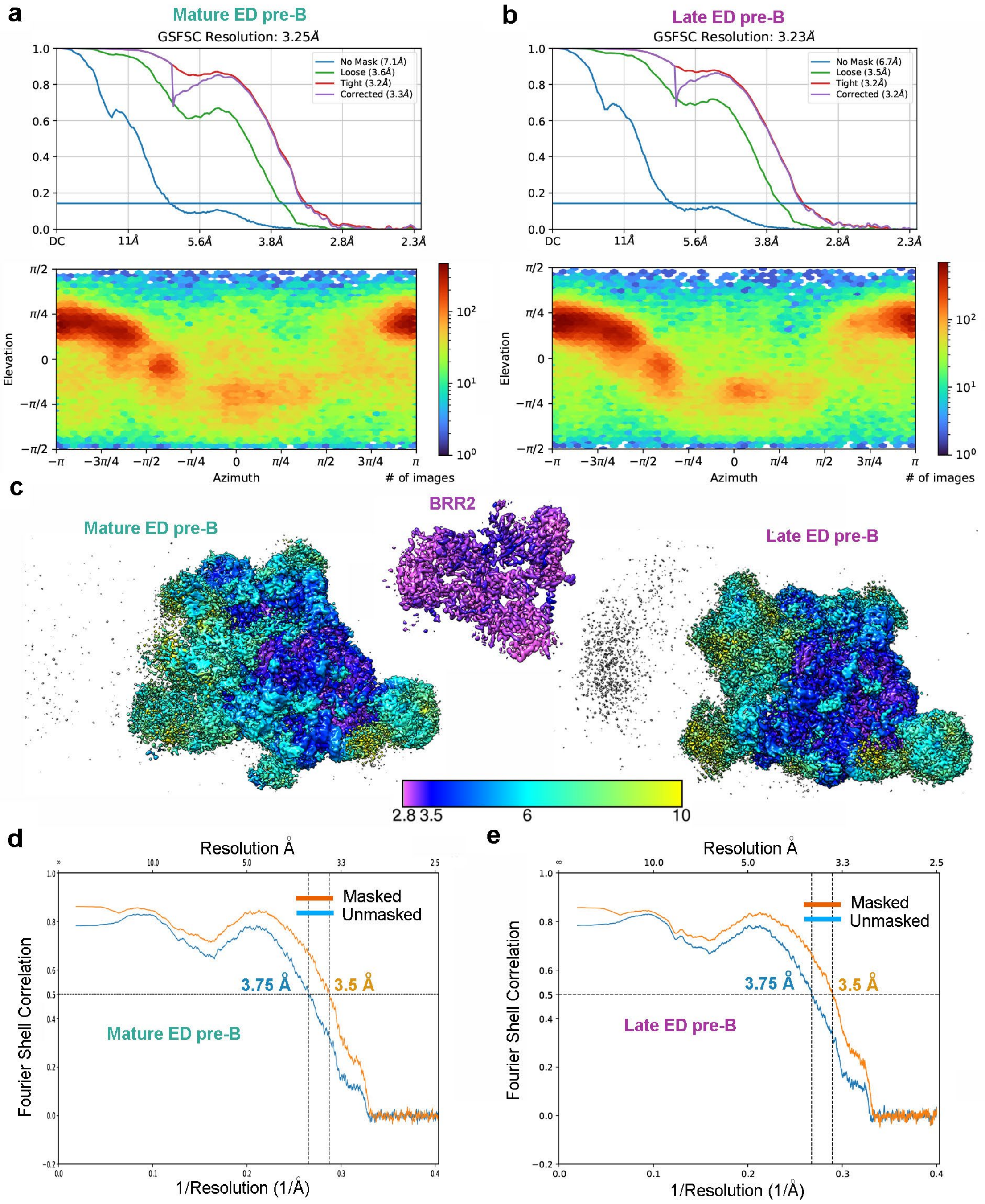

Fig. S3 Cryo-EM analysis and model validation for the human mature ED pre-B and late ED pre-B complexes. **a** The Fourier Shell Correlation (FSC) curve and angular distribution of the particles used for reconstruction of the mature ED pre-B complex. **b** The FSC curve and angular distribution of the particles used for reconstruction of the late ED pre-B complex. **c** The cryo-EM reconstructions of the mature ED pre-B complex (left), the late ED pre-B complex (right), and the focused

refined BRR2 region (middle). The local resolutions of the EM maps are color-coded. **d** The FSC curves of the refined models versus the corresponding maps that are refined against for the mature ED pre-B complex. **e** The FSC curves of the refined models versus the corresponding maps that are refined against for the late ED pre-B complex.
