# Supplementary material for: Structural insights into human exon-defined spliceosome prior to activation
Source: Cell Res. 2024 Apr 24;34(6):428–39. doi: 10.1038/s41422-024-00949-w (PMC11143319; doi:10.1038/s41422-024-00949-w)
Supplement: Supplementary file 4 — Supplementary information, Figure S4 [file 41422_2024_949_MOESM4_ESM.pdf]

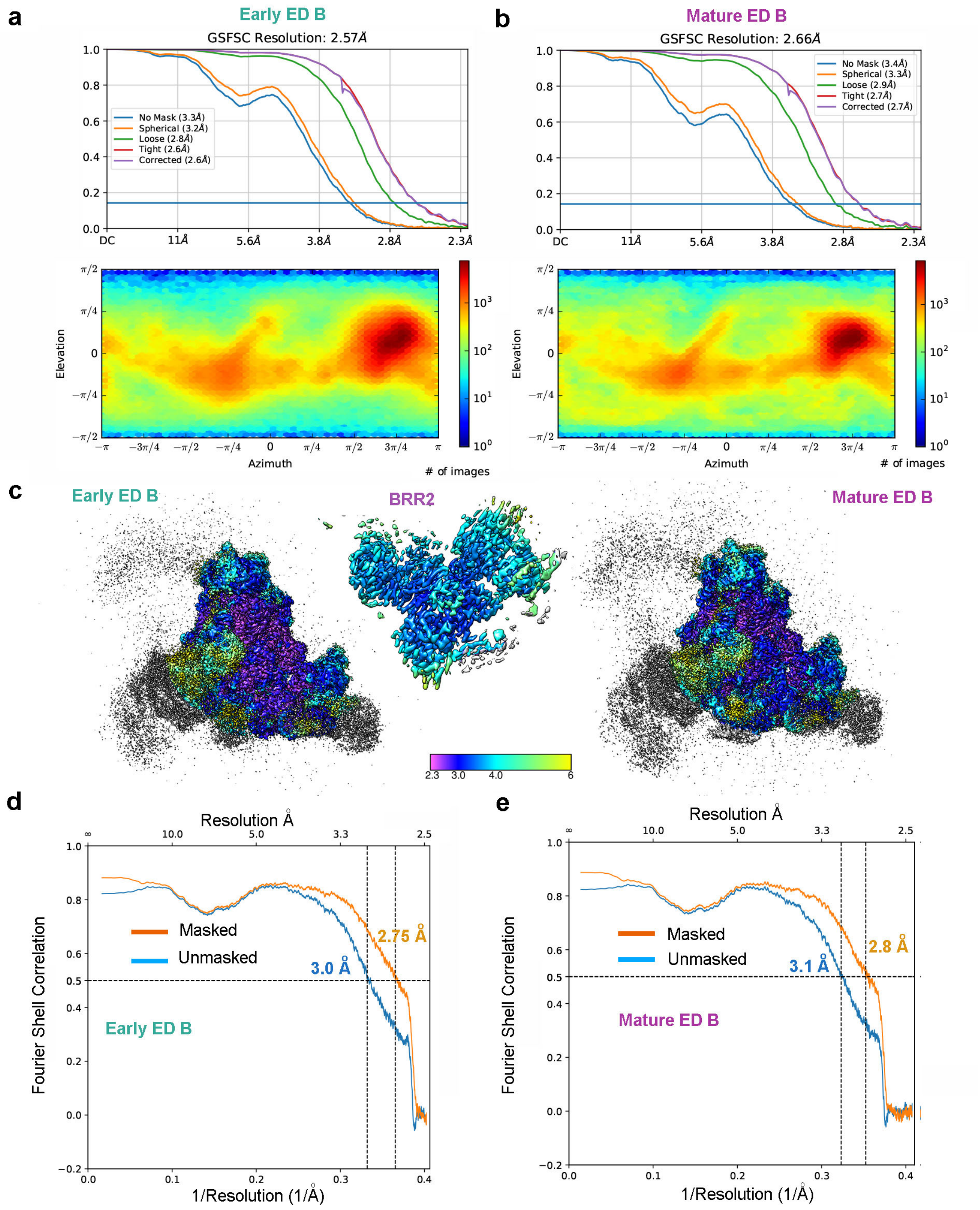

Fig. S4 Cryo-EM analysis and model validation for the human early ED B and mature ED B complexes. **a** The FSC curve and angular distribution of the particles used for reconstruction of the early ED B complex. **b** The FSC curve and angular distribution of the particles used for reconstruction of the mature ED B complex. **c** The cryo-EM reconstructions of the early ED B complex (left), the mature ED B complex (right), and the focused refined BRR2 region (middle). The local resolutions

of the EM maps are color-coded. **d** The FSC curves of the refined models versus the corresponding maps that are refined against for the early ED B complex. **e** The FSC curves of the refined models versus the corresponding maps that are refined against for the mature ED B complex.
