# Supplementary material for: Structural insights into human exon-defined spliceosome prior to activation
Source: Cell Res. 2024 Apr 24;34(6):428–39. doi: 10.1038/s41422-024-00949-w (PMC11143319; doi:10.1038/s41422-024-00949-w)
Supplement: Supplementary file 5 — Supplementary information, Figure S5 [file 41422_2024_949_MOESM5_ESM.pdf]

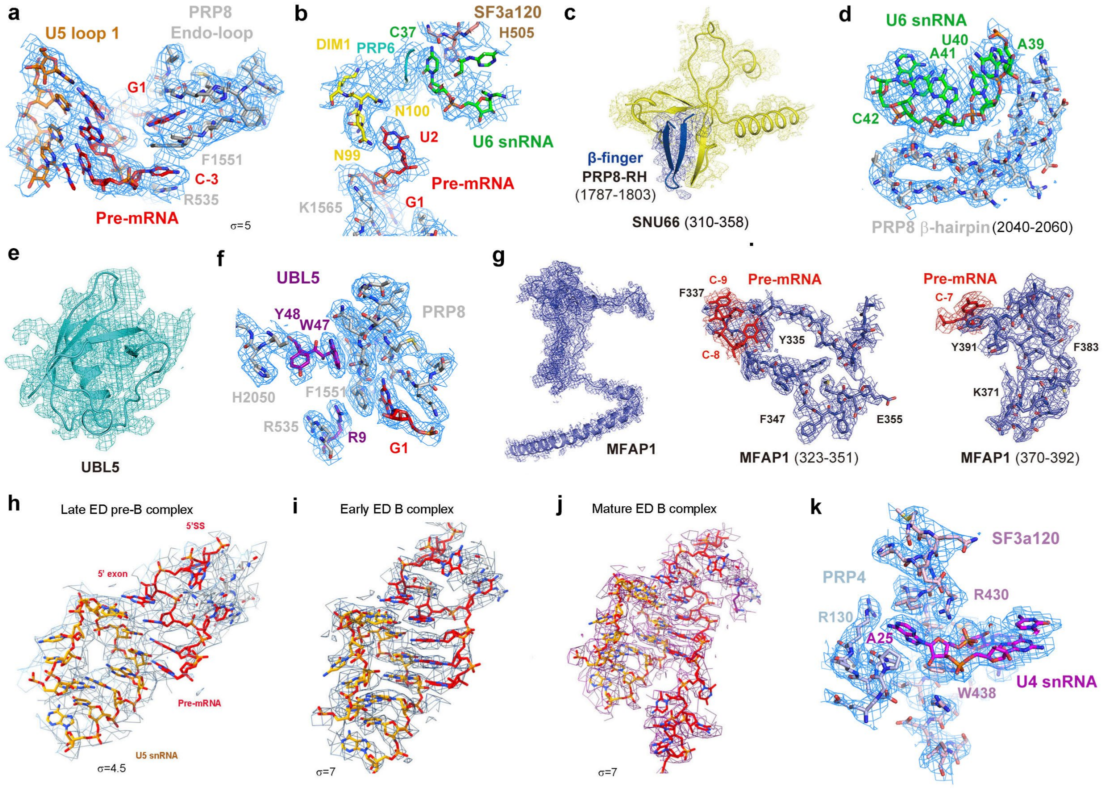

Fig. S5 Representative EM density maps for the newly identified protein sequences and the interface between 5'-exon and U5 loop I. **a** The local EM density map for the double-sandwich structure of pre-mRNA recognition in the late ED pre-B complex. **b** The local EM density map for the interfaces of 5'SS of pre-mRNA/DIM1, and U6 snRNA/PRP6/SF3a120. **c** The EM density map for the interface between SNU66 (yellow) and the β-finger (blue) from the PRP8 RH domain in the mature ED B complex. **d** The local EM density map for the interface between the PRP8 β-hairpin and the U6/5'SS duplex in the early ED B complex. **e** The local EM density map for the double-sandwich structure of pre-mRNA recognition in the mature ED pre-B complex. C-3 of pre-mRNA is replaced by Arg9 of UBL5. **f** The EM density map of UBL5 in the mature ED B complex. **g** The EM density map of MFAP1 in the mature ED B complex. The EM density map for the interfaces

between MFAP1 and the nucleotides C-8 and C-9 or C-7 from the exon are shown in the middle and right panels. **h** The EM density map for the interface between 5'-exon and U5 loop I in the late ED pre-B complex. **i** The EM density map for the interface between 5'-exon and U5 loop I in the early ED B complex. **j** The EM density map for the interface between 5'-exon and U5 loop I in the mature ED B complex. **k** The EM density map for the interface of SF3a120, PRP4 and the nucleotide A25 from U4 snRNA.
