# Supplementary material for: Structural insights into human exon-defined spliceosome prior to activation
Source: Cell Res. 2024 Apr 24;34(6):428–39. doi: 10.1038/s41422-024-00949-w (PMC11143319; doi:10.1038/s41422-024-00949-w)
Supplement: Supplementary file 6 — Supplementary information, Figure S6 [file 41422_2024_949_MOESM6_ESM.pdf]

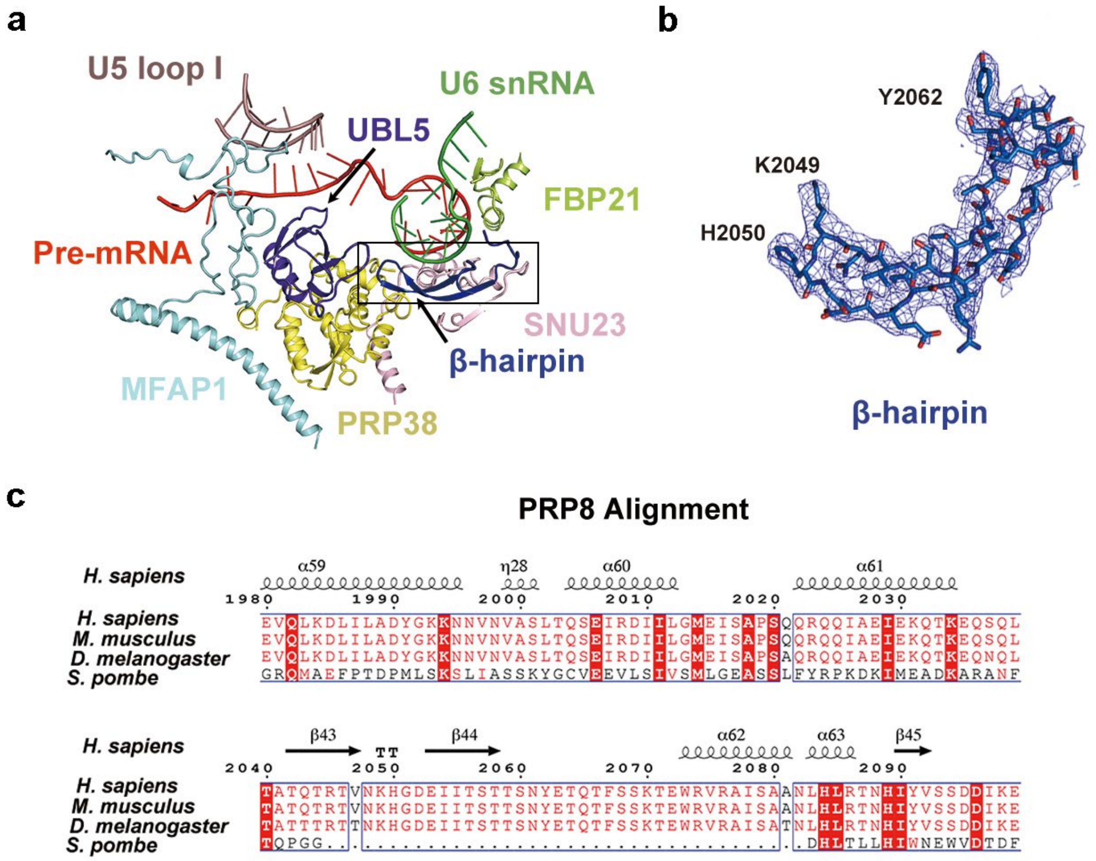


**Fig. S6 The role of the PRP8 β-hairpin in the mature ED B complex.** **a** The newly identified PRP8 β-hairpin appears to play an important role at the center of the mature ED B complex. Shown here is a close-up view on the PRP8 β-hairpin in the core region of the spliceosome. **b** The EM density map of the PRP8 β-hairpin in the mature ED B complex. **c** Sequence alignment of the PRP8 β-hairpin region from different species. The alignment was prepared using an online server (https://espript.ibcp.fr/ESPript/ESPript/) [61].
