# Supplementary material for: Structural insights into human exon-defined spliceosome prior to activation
Source: Cell Res. 2024 Apr 24;34(6):428–39. doi: 10.1038/s41422-024-00949-w (PMC11143319; doi:10.1038/s41422-024-00949-w)
Supplement: Supplementary file 7 — Supplementary information, Figure S7 [file 41422_2024_949_MOESM7_ESM.pdf]

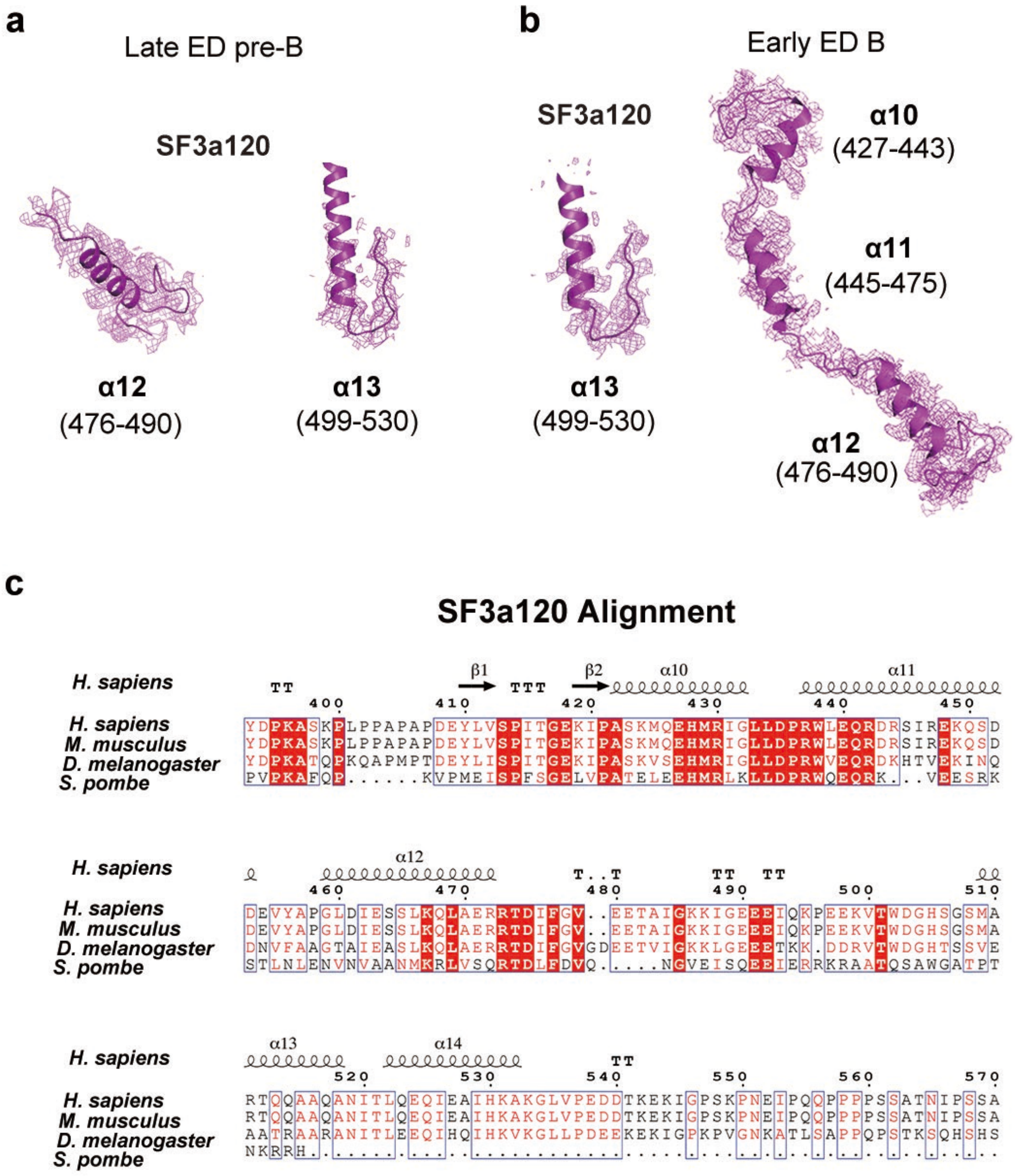

Fig. S7 The SF3a120 fragments that are anchored on the tri-snRNP in the human ED pre-B and ED B complexes. **a** The EM density map for the region of SF3a120 that interacts with the tri-snRNP core domain in the human late ED pre-B complex. **b** The EM density map for the region of SF3a120 that interacts with the tri-snRNP core domain in the human early ED B complex. **c** Sequence alignment of the newly identified region of SF3a120 from different species.
