# Supplementary material for: Structural insights into human exon-defined spliceosome prior to activation
Source: Cell Res. 2024 Apr 24;34(6):428–39. doi: 10.1038/s41422-024-00949-w (PMC11143319; doi:10.1038/s41422-024-00949-w)
Supplement: Supplementary file 8 — Supplementary information, Figure S8 [file 41422_2024_949_MOESM8_ESM.pdf]

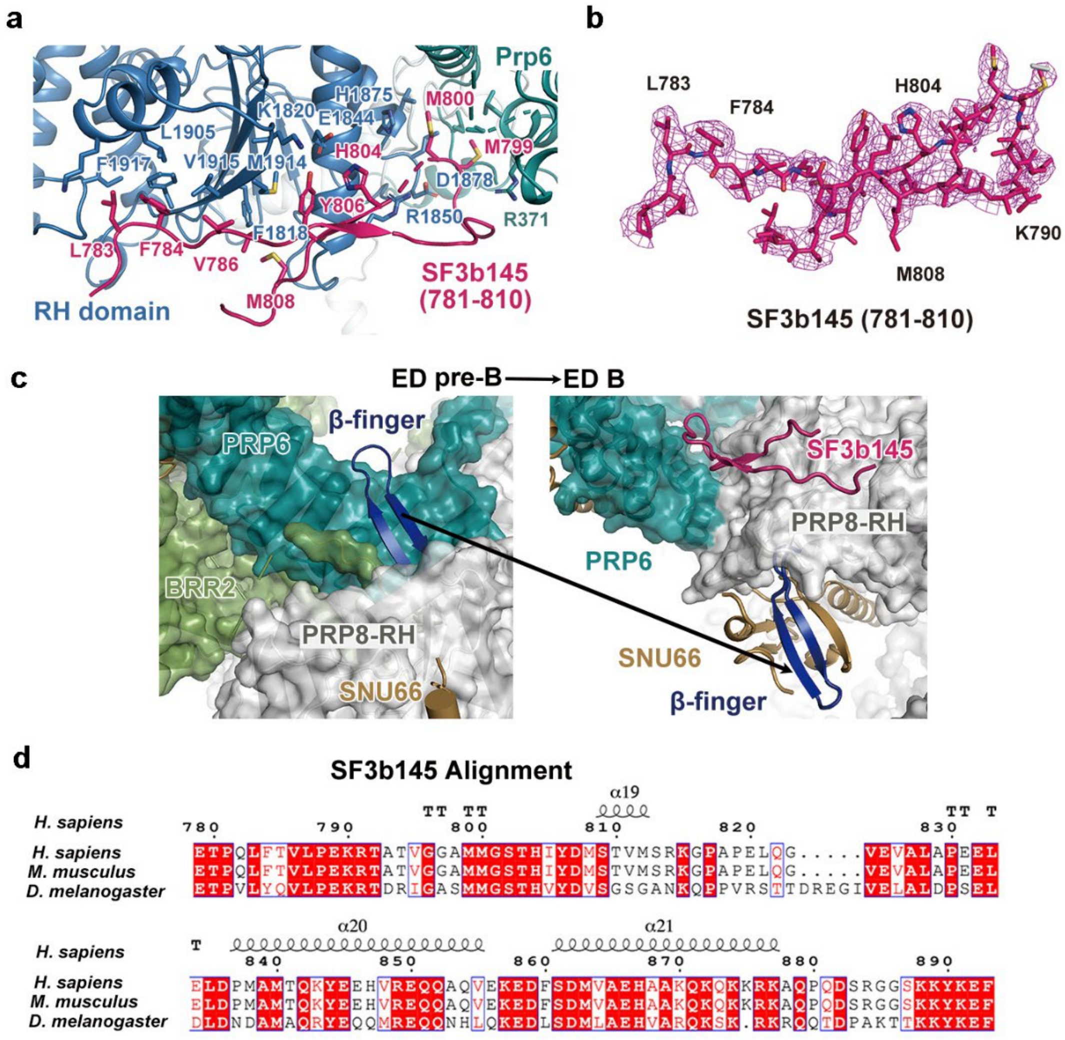

Fig. S8 SF3b145 interacts with the tri-snRNP in the human mature ED B complex. **a** Elements of SF3b145 interact with PRP6 and the RH domain of PRP8 in the tri-snRNP of the mature ED B complex. **b** The EM density map of the newly resolved region of SF3b145 in the mature ED B complex. **c** SF3b145 occupies the same region in the ED B complex as that occupied by the β-finger of the PRP8 RH domain in the ED pre-B complex. SF3b145 also interacts with the PRP8 RH domain and PRP6 HEAT domains in the ED B complex. During the pre-B to B transition, the β-finger is flipped and interacts with SNU66. **d** Sequence alignment of the newly identified region of SF3b145 from different species.
