# Supplementary material for: Structural insights into human exon-defined spliceosome prior to activation
Source: Cell Res. 2024 Apr 24;34(6):428–39. doi: 10.1038/s41422-024-00949-w (PMC11143319; doi:10.1038/s41422-024-00949-w)
Supplement: Supplementary file 9 — Supplementary information, Figure S9 [file 41422_2024_949_MOESM9_ESM.pdf]

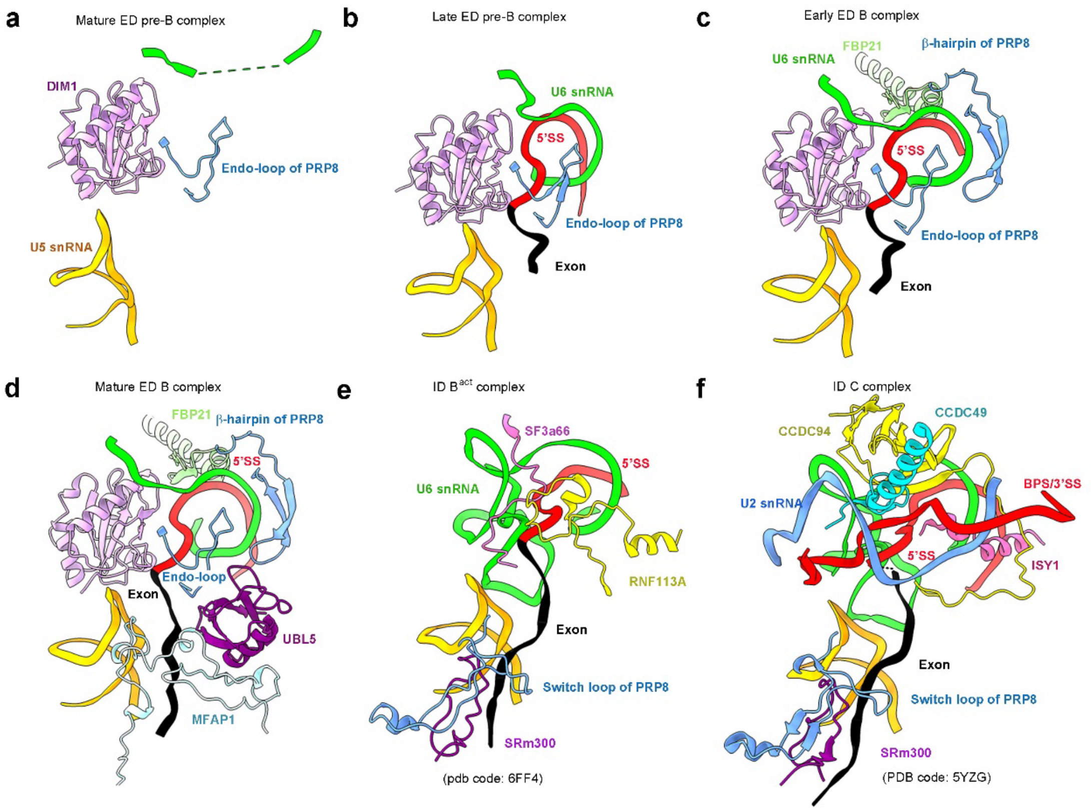

Fig. S9 The interactions between the exon and U5 loop I in different states of the spliceosome. **a** The exon is yet to be loaded onto U5 loop I in the human mature ED pre-B complex. **b** The exon and 5'SS form duplexes with U5 loop I and U6 ACAGA box, respectively, in the human late ED pre-B complex. The Endo-loop of PRP8 stabilizes the 5'SS. The exon has a double-sandwiched structure, which may help reduce the tension associated with its base pairing interactions with U5 loop I. **c** The exon in the human early ED B complex closely resembles that in the late ED pre-B complex. Moreover, the recruitment of FBP21 and the β-hairpin of PRP8 serves to stabilize the U6/5'SS duplex. **d** The exon in the human mature ED B complex. Two B-specific splicing factors, the N-terminal fragments of MFAP1 and UBL5, are recruited to the exon. They help stabilize the interaction between the exon and U5 loop I. **e** The 5'-exon in the human ID B^{act} complex. RNF113A and SF3a66 help stabilize

the U6/5'SS duplex, whereas SRm300 and the switch loop of PRP8 stabilize the U5 loop I/5'-exon duplex. The splicing active site of U6 snRNA is established in the ID B^{act} complex. **f** The 5'-exon in the human ID C complex. SRm300 and the switch loop of PRP8 continue to stabilize the U5 loop I/5'-exon duplex. Additionally, CCDC94, CCDC49, ISY1, and the U2/BPS duplex help stabilize the 5'SS. The first branching reaction has occurred, resulting in a free 5'-exon.
