# Supplementary material for: Structural insights into human exon-defined spliceosome prior to activation
Source: Cell Res. 2024 Apr 24;34(6):428–39. doi: 10.1038/s41422-024-00949-w (PMC11143319; doi:10.1038/s41422-024-00949-w)
Supplement: Supplementary file 10 — Supplementary information, Figure S10 [file 41422_2024_949_MOESM10_ESM.pdf]

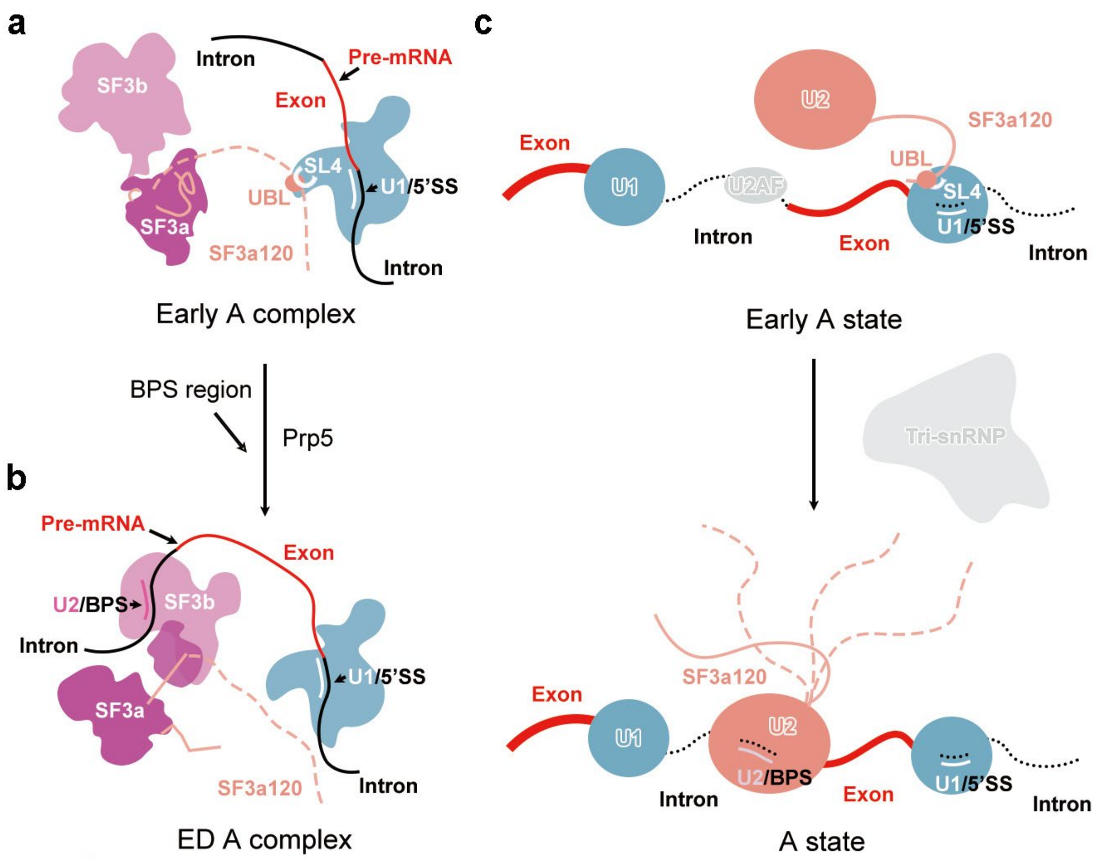

Fig. S10 The transition from the early A complex to A complex. **a** A cartoon model of the early A complex. The C-terminal fragment of SF3a120 from U2 snRNP interacts with the SL4 of U1 snRNP (U1-SL4) [37-39]. **b** A cartoon model of the A complex. The A complex is assembled following BPS recognition by U2 snRNP with the help of PRP5 [16,62]. SF3a120 no longer interacts with U1-SL4 [37]. **c** A cartoon diagram of the transition from the early A to A state. At the early A state (upper panel), U1 snRNP recognizes the 5'SS. U2 snRNP, which is anchored onto U1-SL4 through SF3a120, is floating around U1 snRNP [39]. At the A state (lower panel), U2 snRNP crosses over the short exon and recognizes the upstream BPS region, waiting to recruit tri-snRNP.
