# Supplementary material for: Structural insights into human exon-defined spliceosome prior to activation
Source: Cell Res. 2024 Apr 24;34(6):428–39. doi: 10.1038/s41422-024-00949-w (PMC11143319; doi:10.1038/s41422-024-00949-w)
Supplement: Supplementary file 11 — Supplementary information, Figure S11 [file 41422_2024_949_MOESM11_ESM.pdf]

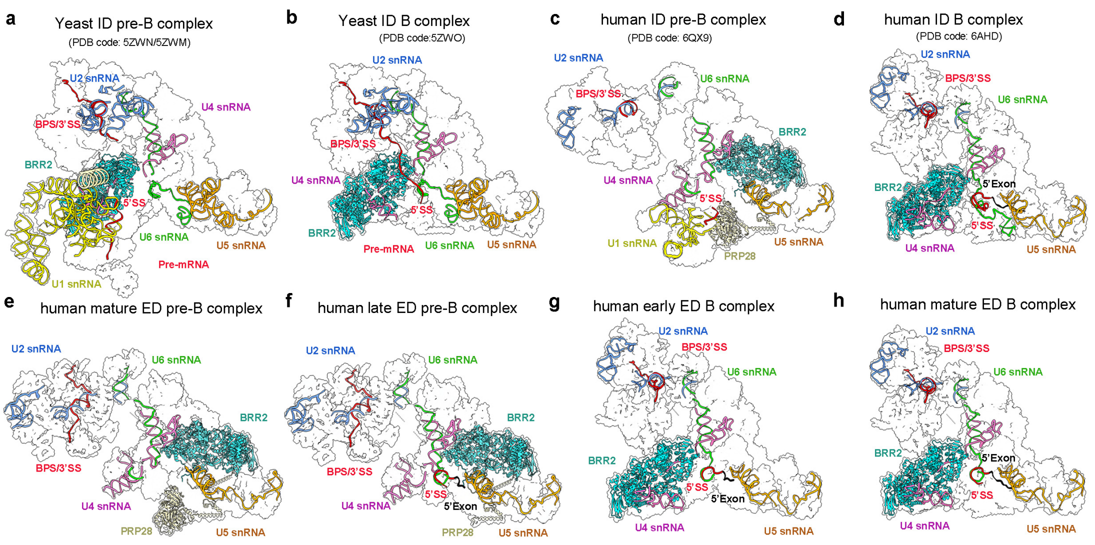

Fig. S11 Spatial organization of the RNA elements in the pre-B and B complexes from both yeast and human. **a** Spatial organization of the RNA elements in the yeast ID pre-B complex. All five snRNAs are present. U1 snRNA forms a duplex with the 5’SS. U6 snRNA base-pairs with U5 loop I, blocking the entry of 5’-exon. **b** Spatial organization of the RNA elements in the yeast ID B complex. U1 snRNA is dissociated by PRP28. At this stage, the 5’SS is yet to base pair with U6 snRNA and the 5’-exon is yet to be delivered into U5 loop I. Throughout the pre-B to B transition in yeast, U5 loop I remains occupied by U6 snRNA. The architecture of the tri-snRNP in yeast ID pre-B and ID B complexes is nearly identical, as indicated by the position of BRR2. U4 snRNA is loaded into the BRR2 helicase. **c** Spatial organization of the RNA elements in the human ID pre-B complex. U1 snRNA forms a duplex with 5’SS. Unlike the yeast pre-B complex, U6 snRNA is not delivered to U5 loop I, and BRR2 occupies a distinct position behind the tri-snRNA without engaging U4 snRNA. **d** Spatial organization of the RNA elements in the human ID B complex. U1 snRNP is dissociated by PRP28. The 5’SS

forms a duplex with U6 snRNA, and the 5'-exon base pairs with U5 loop I. BRR2 is translocated to a position similar to that in yeast ID pre-B and ID B complexes, with U4 snRNA loaded into BRR2. **e** Spatial organization of the RNA elements in the human mature ED pre-B complex. The architecture is similar to that of the human ID pre-B complex, with the exception that U1 snRNP is too flexible to be visualized with wild-type PRP28. **f** Spatial organization of the RNA elements in the human late ED pre-B complex. The overall architecture resembles that of the mature ED pre-B complex. However, U1 snRNP is dissociated by PRP28. The freed 5'SS forms a duplex with U6 snRNA, and the 5'-exon is loaded into U5 loop I. **g** Spatial organization of the RNA elements in the human early ED B complex. The 5'SS forms a duplex with U6 snRNA, and the 5'-exon is loaded into U5 loop I. BRR2 undergoes drastic translocation and is loaded with U4 snRNA. **h** Spatial organization of the RNA elements in the human mature ED B complex. The overall architecture is similar to that of the early ED B complex, with additional B-specific factors recruited to stabilize the complex.
