# Supplementary material for: Structural insights into human exon-defined spliceosome prior to activation
Source: Cell Res. 2024 Apr 24;34(6):428–39. doi: 10.1038/s41422-024-00949-w (PMC11143319; doi:10.1038/s41422-024-00949-w)
Supplement: Supplementary file 12 — Supplementary information, Figure S12 [file 41422_2024_949_MOESM12_ESM.pdf]

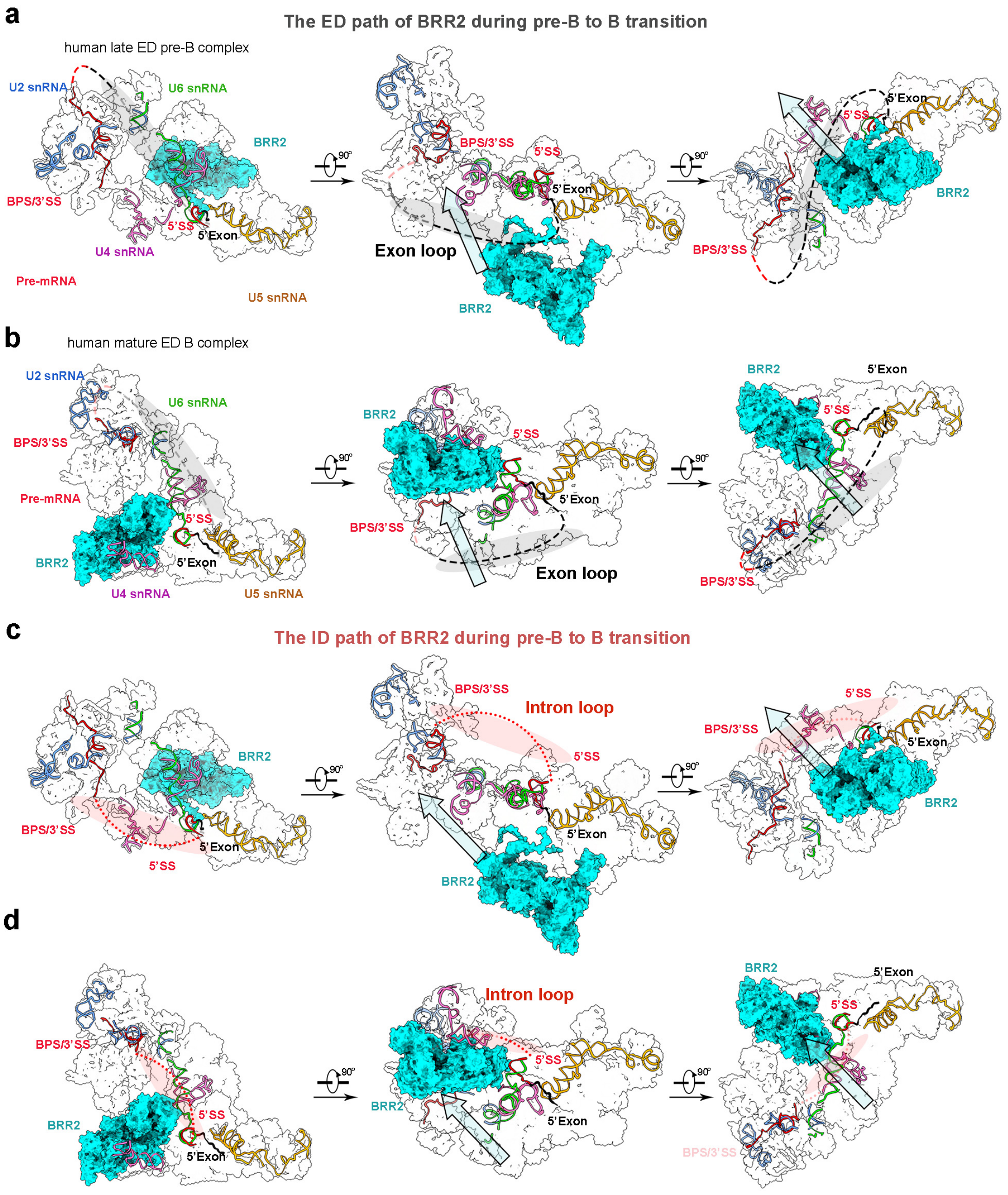

Fig. S12 The ED path for BRR2 translocation during ED pre-B to ED B transition is more restrictive than the ID path. **a** The ED path in the human late ED pre-B complex. The ED path represents the trajectory of BRR2 translocation during the transition from the ED pre-B complex to ED B complex. The spatial loop formed by the exon acts as a topological obstacle for BRR2 translocation. Three mutually perpendicular views are shown here. **b** The ED path in the mature ED B

complex. **c** The ID path in the human ID pre-B complex. The ID path represents the trajectory of BRR2 translocation during the transition from the ID pre-B complex to the ID B complex. The spatial loop formed by the intron has no impact on BRR2 translocation. **d** The ID path in the human ID B complex.
