# Supplementary material for: Structural insights into human exon-defined spliceosome prior to activation
Source: Cell Res. 2024 Apr 24;34(6):428–39. doi: 10.1038/s41422-024-00949-w (PMC11143319; doi:10.1038/s41422-024-00949-w)
Supplement: Supplementary file 13 — Supplementary information, Figure S13 [file 41422_2024_949_MOESM13_ESM.pdf]

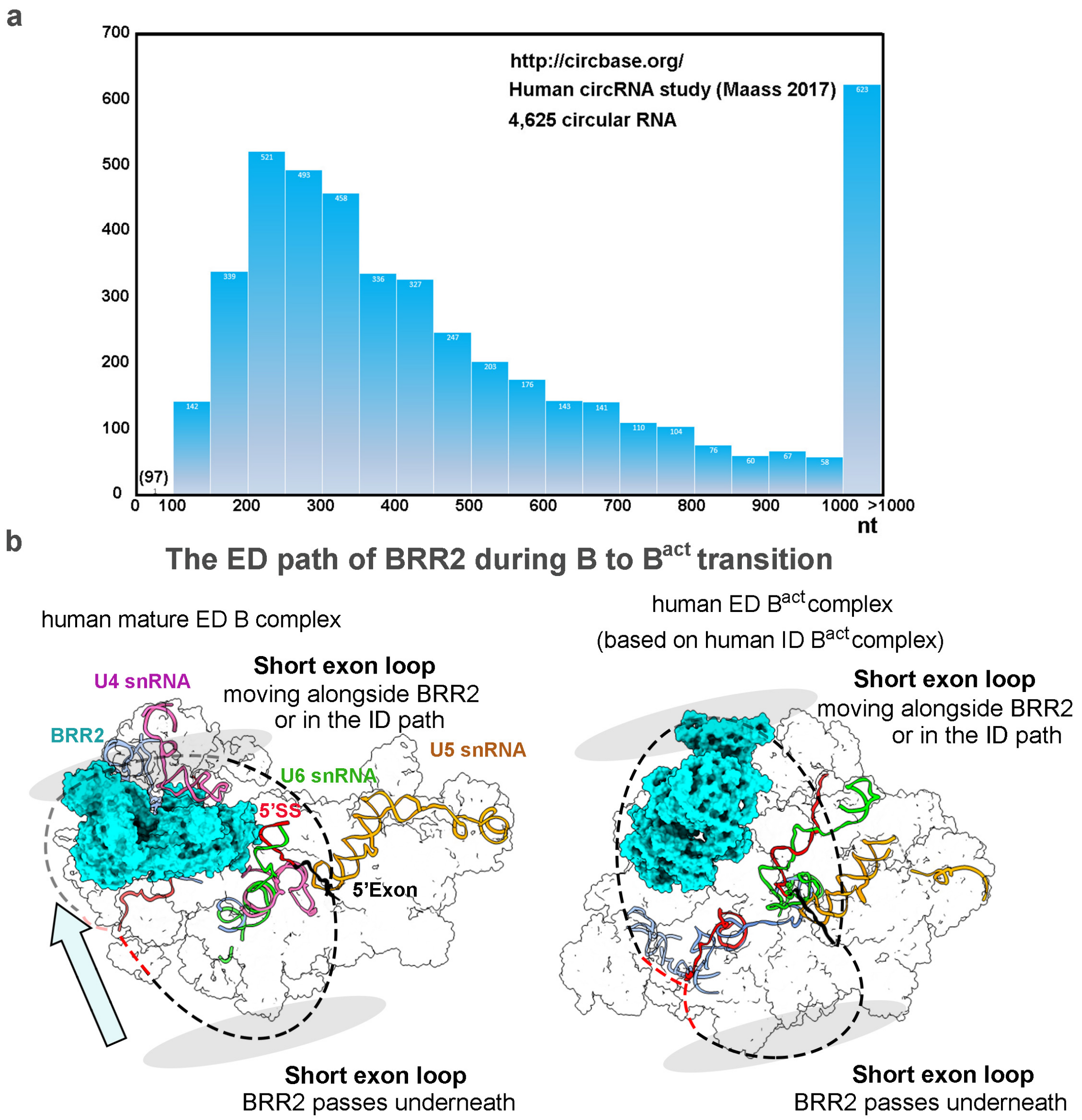

Fig. S13 Analysis of circular RNA generation. **a** The length of circular RNA is concentrated in the range of 150-500 nucleotides. A dataset of 4,625 circular RNAs was employed for statistical analysis. This dataset is available at http://circbase.org/.

b Two potential ED paths for BRR2 translocation during the transition from the ED B complex to ED B^{act} complex. If the short exon moves alongside BRR2 during the pre-B to B transition or just occupying the ID path during pre-B formation (upper ED path), it may act as a topological obstacle for BRR2 translocation during the ED B to ED B^{act} transition. This process requires a longer exon. If BRR2 successfully traverses

underneath the exon loop (lower ED path), the ED B to ED B^{act} transition is topologically permissible.
