# Supplementary material for: Structural insights into human exon-defined spliceosome prior to activation
Source: Cell Res. 2024 Apr 24;34(6):428–39. doi: 10.1038/s41422-024-00949-w (PMC11143319; doi:10.1038/s41422-024-00949-w)
Supplement: Supplementary file 14 — Supplementary information, Figure S14 [file 41422_2024_949_MOESM14_ESM.pdf]

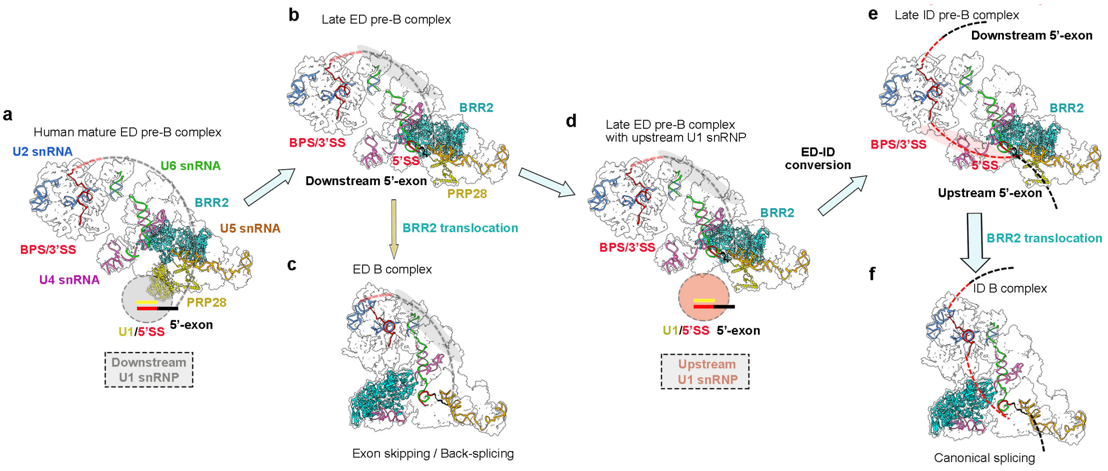

Fig. S14 A working model of U1 swapping during the ED-to-ID conversion. **a** Formation of the human mature ED pre-B complex. The downstream U1 snRNP are indicated by a gray circle. U1 snRNA, 5'SS, and 5'-exon are represented by yellow, red, and black lines, respectively. **b** Formation of the late ED pre-B complex. U1 snRNP is dissociated by PRP28. The freed 5'SS forms a duplex with U6 snRNA, and the 5'-exon is delivered into U5 loop I. Importantly, at this stage, a short exon may hinder BRR2 translocation. Such hindrance results in an increase of the residence time of the spliceosome in the late ED pre-B state. **c** Formation of the late ED B complex. If BRR2 manages to bypass the hindrance posed by the exon loop in the ED path, the late ED pre-B complex will be remodeled into the ED B complex, in which back-splicing occurs. **d** Recruitment of upstream U1 snRNP to the late ED pre-B complex. If the spliceosome is stalled in the late ED pre-B state, the upstream U1 snRNP can be recruited into the complex. The upstream U1 snRNP is indicates by an orange circle. **e** The ED-to-ID conversion. PRP28 again acts on the upstream U1 snRNP and frees the upstream 5'SS and 5'-exon, which, in turn, compete with the loosely assembled downstream 5'SS and 5'-exon, forming the late ID pre-B complex.

f Formation of ID B complex. The conversion of the late ED pre-B complex to the late ID pre-B complex results in a shift from the ED path to the ID path. This transition enables BRR2 translocation, leading to formation of the ID B complex.
