# Supplementary material for: Structural insights into human exon-defined spliceosome prior to activation
Source: Cell Res. 2024 Apr 24;34(6):428–39. doi: 10.1038/s41422-024-00949-w (PMC11143319; doi:10.1038/s41422-024-00949-w)
Supplement: Supplementary file 15 — Supplementary information, Table S1 [file 41422_2024_949_MOESM15_ESM.pdf]

Table S1. Cryo-EM data collection, refinement and validation statistics of the ED spliceosomes.

	Mature ED Pre-B	Late ED Pre-B	Early ED B	Mature ED B
**Data collection and processing**				
Magnification	64,000			
Voltage (kV)	300			
Electron exposure ($e^-/Å^2$)	50			
Defocus range (μm)	-1.5 to -2.0			
Pixel size (Å)	1.10			
Symmetry imposed	C1			
Raw movies	37,699			
Particle Number	116,801	140,426	716,083	414,060
Map resolution (Å)	3.2	3.2	2.6	2.7
FSC threshold	0.143			
Map resolution range (Å)	40~2.8	40~2.8	40~2.4	40~2.4
**Refinement (Core region)**				
Model resolution (FSC=0.5)	3.5	3.5	2.75	2.8
Protein residues (all)	14,606	14,318	13,924	14,409
RNA (all)	444	466	468	476
Protein residues (Core region)	6,304	6,399	7,495	7,930
RNA (Core region)	104	135	206	214
Ligands	5	5	6	6
B factors ($Å^2$)				
Protein	108.02	99.45	31.58	32.40
RNA	169.21	118.51	34.88	38.75
Ligand	67.82	50.77	58.64	45.18
R.m.s. deviations				
Bond lengths (Å)	0.003	0.004	0.003	0.004
Bond angles (°)	0.573	0.610	0.614	0.628
Validation				
MolProbity score	1.72	1.73	2.08	1.99
Clashscore	7.94	7.55	4.88	5.38
Ramachandran plot				
Favored (%)	95.79	95.45	93.86	94.71
Allowed (%)	4.19	4.47	6.11	5.20
Disallowed (%)	0.02	0.08	0.04	0.09
Correct sugar puckers (%)	95.20	94.74	97.96	98.04
Average suiteness (%)	0.557	0.531	0.536	0.546
**PDB code**	8H6J	8H6E	8H6L	8H6K
**EMDB code**	EMD-34505	EMD-34500	EMD-34508	EMD-34507
