# Supplementary material for: Structural insights into human exon-defined spliceosome prior to activation
Source: Cell Res. 2024 Apr 24;34(6):428–39. doi: 10.1038/s41422-024-00949-w (PMC11143319; doi:10.1038/s41422-024-00949-w)
Supplement: Supplementary file 16 — Supplementary information, Table S2 [file 41422_2024_949_MOESM16_ESM.pdf]

Table S2. Summary of model building statistics for the human mature ED and late ED pre-B complexes.

		Molecule<br>Human/*S.pombe*/*S.cere*	Length	Domain/Region	PDB code	Modeling	Resolution (Å)	Chain ID
**U5 snRNP**	Mature & Late	U5 snRNA	117	4:117	6AH0	DR	2.8~5.0	5A
		PRP8/Spp42/Prp8	2335	25:51/58:663/675:2016/2067:2335		DR	2.8~5.0	5B
		SNU114/Cwf10/Snu114	972	105:956		DR	2.8~5.0	5C
		BRR2	2136	36:223/405:2125		DR	2.8~5.0	5D
		U5-40K/Cwf17/-	357	WD40 domain		RD	8.0~10.0	5E
		SmB,D1,D2,D3,E,F,G	-	Sm fold		RD	4.0~10.0	5a-g
**U6 snRNP**	**Mature**<br>**Late**	U6 snRNA	107 nt	31:36/47:78/85:105<br>36:78/85:105	6AH0 & 6QX9	DR<br>HM	2.8~6.0	6A
	Mature & Late	Lsm2-8	-	Sm fold		RD	20.0~30.0	6a-g
**U4 snRNP**	Mature & Late	U4 snRNA	144 nt	1:145	6AH0 & 6QX9	HM	2.8~8.0	4A
		SmB,D1,D2,D3,E,F,G	-	Sm fold		RD	5.0~8.0	4a-g
**Tri-snRNP Core proteins**	Mature & Late	PRP3	683	Ferredoxin-like domain	6AH0 & 6QX9	RD	4.0~8.0	4B
		PRP4/Cwf3/Syf1	522	WD40 domain		RD	4.0~8.0	4C
		PRP31/Cwf7/Snt309	499	Nop domain 52:432		RD	4.0~8.0	4D
		SNU13/Cdc5/Cef1	128	5:128		RD	4.0~8.0	4E
		DIM1/Dim1/Dib1	142	Thioredoxin-like		DR	2.8~3.0	4F
		PRP6/Cwf4/Clf1	941	NTD; TPR repeat		RD	2.8~8.0	4G
		SNU66	800	699:774		DR	3.5~5.0	4S
**Pre-B specific proteins**	Mature & Late	RBM42/-/-	480	RRM domain	6QX9	DR	3.5~5.0	4R
		USP39/Sad1/Sad1	565	103:565		DR	4.0~8.0	4T
		U4/U6.U5-27K/-/-	155	131:151		DR	3.5~5.0	4X
		PRP4 kinase/-/Prp4 kinase	1007	Kinase domain		RD	20.0~30.0	4Y
	**Mature**<br>**Late**	DDX23/Prp28/Prp28	820	N-plug/Catalytic domain<br>N-plug		DR	2.8~5.0	4U
**U2 snRNP**	Mature & Late	U2 RNA	188 nt	1:47/54:184	6AH0 & 6QX9	RD	20.0~30.0	2A
		U2-A'/Lea1/Lea1	255	LRR domain		RD	20.0~30.0	2B
		U2-B"/Msl1/Msl1	225	RRM domain		RD	20.0~30.0	2C
		SmB,D1,D2,D3,E,F,G	-	Sm fold		RD	20.0~30.0	2a-g
	**Mature**<br>**Late**	SF3a120/Sap114/Prp21	793	160:285<br>160:285/455:488/499:503		RD<br>HM	3.0~30.0	2D
	Mature & Late	SF3a66/Sap62/Prp11	464	92:233		RD	20.0~30.0	2E
		SF3a60/Sap61/Prp9	501	1:374/390:463		RD	20.0~30.0	2F
		SF3b155/Sap155/Hsh155	1304	HEAT repeat		RD	20.0~30.0	2G
		SF3b145/Sap145/Cus1	895	461:600/604:692		RD	3.0~30.0	2H
		SF3b130/Sap130/Rse1	1217	WD40 domain I/II/III		RD	20.0~30.0	2I
		SF3b49/Sap49/Hsh49	424	RRM domain I/II		RD	20.0~30.0	2J
		SF3b14a/p14-like/-	125	RRM domain		RD	20.0~30.0	2K
		SF3b14b/Ini1/Rds3	110	PHF5 domain		RD	20.0~30.0	2L
		SF3b10/SF3b10/Ysf3	86	15:80		RD	20.0~30.0	2M
**Pre-mRNA**	**Mature**<br>**Late**	Pre-mRNA	144 nt	7:48 nt<br>7:48/103:119 nt	6AH0	RD/HM	3.0~20.0	A
**U1 snRNP**	**Mature**<br>**Late**	U1 snRNP molecules	-	Attached<br>Released	-	Not modeled	-	-
