# Supplementary material for: Structural insights into human exon-defined spliceosome prior to activation
Source: Cell Res. 2024 Apr 24;34(6):428–39. doi: 10.1038/s41422-024-00949-w (PMC11143319; doi:10.1038/s41422-024-00949-w)
Supplement: Supplementary file 17 — Supplementary information, Table S3 [file 41422_2024_949_MOESM17_ESM.pdf]

Table S3. Summary of model building statistics of the human early ED and mature ED B complexes.

		Molecule<br>Human/*S.pombe*/*S.cere*	Length	Domain/Region	PDB code	Modeling	Resolution (Å)	Chain ID
**U5 snRNP**	Early & Mature	U5 snRNA	117	3:116	6AHD	DR	2.3~5.0	5A
		PRP8/Spp42/Prp8	2335	56:662/675:2026/2067:2335		DR	2.3~5.0	5B
		SNU114/Cwf10/Snu114	972	112:943		DR	2.3~5.0	5C
		BRR2	2136	404:2125		DR	3.0~4.0	5D
		U5-40K/Cwf17/-	357	WD40 domain		RD	15.0~30.0	5E
		SmB,D1,D2,D3,E,F,G	-	Sm fold		RD	4.0~15.0	5a-g
**U6 snRNP**	Early & Mature	U6 snRNA	107 nt	1:97	6AHD	DR	2.3~6.0	6A
		Lsm2-8	-	Sm fold		RD	15.0~30.0	6a-g
**U4 snRNP**	Early & Mature	U4 snRNA	144 nt	1:145	6AHD	DR	2.3~8.0	4A
		SmB,D1,D2,D3,E,F,G	-	Sm fold		RD	5.0~8.0	4a-g
**Tri-snRNP specific proteins**	Early & Mature	PRP3	683	Ferredoxin-like domain	6AHD	DR	3.0~5.0	4B
		PRP4/Cwf3/Syf1	522	WD40 domain		DR	3.0~5.0	4C
		PRP31/Cwf7/Snt309	499	Nop domain 52:432		DR	3.0~5.0	4D
		SNU13/Cdc5/Cef1	128	5:128		DR	2.5~3.0	4E
		SNU66/-/-	800	146:214/248:358		DR/HM	3.0~4.0	4J
		DIM1/Dim1/Dib1	142	Thioredoxin-like		DR	2.3~3.0	5F
		PRP6/Cwf4/Clf1	941	NTD; TPR repeat		DR	2.5~8.0	5G
**B specific**	Early & Mature	CYPH/CypH/-	177	Cyclophilin domain	6AHD	DR	3.5~5.0	4H
		FBP21/-/-	376	8:82		DR	3.5~5.0	4I
		SMU1/-/-	513	WD40 domain		RD	10.0~20.0	4Z
	**Mature**	PRP38	312	2:176		DR	2.5~4.0	4L
		UBL5/-/Hub1	73	Ubiquitin like domain		DR/HM	2.3~3.0	4M
		SNU23	199	55:134		DR	3.0~4.0	4N
		MFAP1/-/Spp381	439	207:254; 270:406		DR/HM	2.5~4.0	4K
**U2 snRNP**		U2 RNA	188 nt	1:47/54:184	6AHD	RD	8.0~20.0	2A
		U2-A'/Lea1/Lea1	255	LRR domain		RD	20.0~30.0	2B
		U2-B"/Msl1/Msl1	225	RRM domain		RD	20.0~30.0	2C
		SmB,D1,D2,D3,E,F,G	-	Sm fold		RD	20.0~30.0	2a-g
	**Early**<br>**Mature**	SF3a120/Sap114/Prp21	793	160:294/411:490/499:530<br>160:294/411:490		RD/HM	20.0~30.0	2D
	Early & Mature	SF3a66/Sap62/Prp11	464	92:233		RD	20.0~30.0	2E
		SF3a60/Sap61/Prp9	501	1:374/390:463		RD	20.0~30.0	2F
		SF3b155/Sap155/Hsh155	1304	HEAT repeat		RD	20.0~30.0	2G
		SF3b145/Sap145/Cus1	895	461:600/604:692/781:811		RD/HM	20.0~30.0	2H
		SF3b130/Sap130/Rse1	1217	WD40 domain I/II/III		RD	20.0~30.0	2I
		SF3b49/Sap49/Hsh49	424	RRM domain I/II		RD	20.0~30.0	2J
		SF3b14a/p14-like/-	125	RRM domain		RD	20.0~30.0	2K
		SF3b14b/Ini1/Rds3	110	PHF5 domain		RD	20.0~30.0	2L
		SF3b10/SF3b10/Ysf3	86	15:80		RD	20.0~30.0	2M
**Pre-mRNA**	**Early**<br>**Mature**	Pre-mRNA	144	7:48/102:116 nt<br>7:48/98:119 nt	6AHD	RD/HM	2.5~30.0	A
